# A lab-on-a-disc platform enables serial monitoring of individual CTCs associated with tumor progression during EGFR-targeted therapy for patients with NSCLC

**DOI:** 10.7150/thno.44693

**Published:** 2020-04-06

**Authors:** Minji Lim, Juhee Park, Alarice C. Lowe, Hyoung-oh Jeong, Semin Lee, Hee Chul Park, Kyusang Lee, Gwang Ha Kim, Mi-Hyun Kim, Yoon-Kyoung Cho

**Affiliations:** 1 Center for Soft and Living Matter, Institute for Basic Science (IBS), Ulsan 44919, Republic of Korea; 2 Department of Biomedical Engineering, School of Life Sciences, Ulsan National Institute of Science and Technology (UNIST), Ulsan 44919, Republic of Korea; 3 Department of Pathology, Stanford University, Stanford, CA, 94305, USA; 4 Clinomics Inc., Ulsan 44919, Republic of Korea; 5 Department of Internal Medicine, Pusan National University School of Medicine and Biomedical Research Institute, Pusan National University Hospital, 179, Gudeok-ro, Seo-Gu, Busan 49241, Republic of Korea

**Keywords:** circulating tumor cells, single cell analysis, gene expression, EGFR mutation, non-small cell lung cancer

## Abstract

**Rationale**: Unlike traditional biopsy, liquid biopsy, which is a largely non-invasive diagnostic and monitoring tool, can be performed more frequently to better track tumors and mutations over time and to validate the efficiency of a cancer treatment. Circulating tumor cells (CTCs) are considered promising liquid biopsy biomarkers; however, their use in clinical settings is limited by high costs and a low throughput of standard platforms for CTC enumeration and analysis. In this study, we used a label-free, high-throughput method for CTC isolation directly from whole blood of patients using a standalone, clinical setting-friendly platform.

**Methods**: A CTC-based liquid biopsy approach was used to examine the efficacy of therapy and emergent drug resistance via longitudinal monitoring of CTC counts, DNA mutations, and single-cell-level gene expression in a prospective cohort of 40 patients with epidermal growth factor receptor (EGFR)-mutant non-small cell lung cancer.

**Results**: The change ratio of the CTC counts was associated with tumor response, detected by CT scan, while the baseline CTC counts did not show association with progression-free survival or overall survival. We achieved a 100% concordance rate for the detection of *EGFR* mutation, including emergence of T790M, between tumor tissue and CTCs. More importantly, our data revealed the importance of the analysis of the epithelial/mesenchymal signature of individual pretreatment CTCs to predict drug responsiveness in patients.

**Conclusion**: The fluid-assisted separation technology disc platform enables serial monitoring of CTC counts, DNA mutations, as well as unbiased molecular characterization of individual CTCs associated with tumor progression during targeted therapy.

## Introduction

Lung cancer is the most common cause of cancer-related mortality in both men and women worldwide 1, 2. Non-small cell lung cancer (NSCLC) accounts for 84% of all lung cancers, and despite improved diagnostic techniques, a great majority of patients with NSCLC present with advanced-stage tumors at diagnosis, and the 5-year relative survival rate is less than 20% 3. Epidermal growth factor receptor (EGFR) mutations are found in 10-30% of patients with NSCLC, and targeted therapies, including EGFR-tyrosine kinase inhibitors (TKIs) can deliver significantly improved clinical outcomes, depending upon the type of mutation 4, 5. As new-generation EGFR-TKIs (e.g. osimertinib) 6, 7 have been developed recently for patients who showed resistance to conventional EGFR-TKIs, frequent examination of the tumor susceptibility to EGFR-TKIs became very important. However, conventional tissue biopsy is invasive, and therefore, more facile and less invasive liquid biopsy methods, able to monitor the disease response to targeted therapy and track the emergence of drug resistance, could significantly aid in the clinical management of the disease.

Among various circulating biomarkers, circulating tumor cells (CTCs) offer a unique opportunity to obtain information on live tumor cells at the DNA, RNA, and protein levels. Despite significant challenges associated with the extremely rare and heterogeneous characteristics of CTCs, recent technological advancements have allowed studying the prognostic value of CTCs, as well as monitoring and predicting the efficacy of therapy. To date, various platforms have been developed to isolate CTCs based on their biochemical or physical properties, such as the expression of epithelial cell adhesion molecule (EpCAM) or their size. Nevertheless, only the CellSearch™ system (Menarini Silicon Biosystems) is currently approved by the US Food and Drug Administration for the enumeration of CTCs in patients with metastatic colorectal 8, breast 9, and prostate 10 cancer. However, the prognostic value of CTCs remains controversial in NSCLC. Although many previous studies have demonstrated that patients with a higher number of CTCs have a poor clinical outcome 11-16, some studies have reported that baseline CTC counts had no significant correlation with the survival rate in patients with NSCLC 17, 18.

Previous immunoaffinity-based CTC enrichment methods using anti-EpCAM antibodies suffer from the limitation of missing clinically important CTCs with low EpCAM expression such as mesenchymal and stem cell-like tumor cells. Furthermore, immunoaffinity-based isolation methods require a relatively long processing time to achieve an efficient antigen-antibody interaction 19, 20. Alternatively, marker-independent approaches, based on intrinsic physical properties of CTCs, have been developed 21, 22, but they tend to show lower capture yields and purity, and sample pretreatment requirements or clogging issues are often a problem. Size-based CTCs isolation methods using inertial microfluidics provide relatively high detection rates but require prior sample preparation steps such as removal of red blood cells and dilution 23, 24. While most of the current CTC enumeration technologies rely on signal averaging across individual heterogeneous CTCs, facile analysis of CTCs at a single-cell resolution is highly desired to assess the cell heterogeneity and uncover its clinical consequences. Although much progress has been made and clinical significance of CTCs has been demonstrated, the remaining challenges include a high cost, low throughput, and complexity of the process, as well as false-positive/false-negative results, which hinder a wider adoption of CTC-based liquid biopsy as a routine practice in clinical settings.

In this report, we present the preclinical validation of a fluid-assisted separation technology (FAST) disc 25, which allows rapid (>3 mL/min), reproducible, and label-free isolation of CTCs directly from unprocessed whole blood of patients with NSCLC. We performed serial monitoring of CTC counts, mutation detection, and single-cell multiplex gene expression of CTCs from a prospective cohort of patients with NSCLC receiving EGFR-TKIs treatment. The study highlights the potential of a CTC-based liquid biopsy for assessing the efficacy of therapy and emergent drug resistance.

## Methods

### Study design and blood sample collection

The biospecimens and data used in this study were provided by the Biobank of the Pusan National University Hospital (PNUH), a member of the Korea Biobank Network. The study protocol was reviewed and approved by the Institutional Review Board (IRB) of PNUH (H1612-019-049). This was a prospective, single-center study conducted at PNUH (Busan, Republic of Korea). Patients who met the following inclusion criteria were selected for the study: 1) a histologically or cytologically confirmed diagnosis of primary NSCLC; 2) a confirmed *EGFR* mutation in histological or cytological specimens; 3) previously untreated stage IIIB or IV according to the 7th edition of TNM staging system by the international association for the study of lung cancer [measurable disease according to Response Evaluation Criteria in Solid Tumors version 1.1 (RECIST 1.1)]; and 4) received at least one dose of EGFR-TKIs as the first-line therapy during the follow-up study. Blood samples were collected for analysis within 3 days before/after EGFR-TKIs treatment. Written informed consent was obtained from all patients before blood sampling.

To monitor CTC counts during TKI therapy, blood samples were collected from 40 enrolled patients on a regular schedule. Peripheral blood (3 mL) was collected in a Vacutainer® (K2 EDTA, #367844; BD Medical) and CTC isolation was completed within 6 h 26-28. For the detection of *EGFR* mutations and single-cell-level gene expression analysis, additional 3 mL blood samples were collected from 16 patients. For serial monitoring of single-cell RNA expression in CTCs, blood samples were collected from 75-year-old male (LP25) and 74-year-old female (LP38) patients diagnosed with 19del and L861Q EGFR mutant-positive lung cancer, respectively, who were receiving afatinib treatment.

### Cell culture

Breast cancer (MCF7, MDA-MB-231, and SKBR3), prostate cancer (LNCaP and PC3), and lung cancer (H460, H2228, HCC78, and PC9) cell lines were purchased from the American Type Culture Collection. All cell lines were cultured in RPMI 1640 medium supplemented with 5% FBS and 1% antibiotics/antimycotics at 37°C, 5% CO_2_. The *mycoplasma* test was confirmed by e-Myco^TM^ Mycoplasma detection kit (Intron, Korea).

### Immunofluorescent staining and analysis of CTCs

Immunofluorescent staining and detection were performed on the FAST disc membrane according to a previously reported protocol 25. Briefly, CTCs captured on a FAST disc were fixed with 4% formaldehyde and permeabilized with 0.1% Triton X-100, followed by washing and blocking with 10 µg/mL IgG in PBS. Cells were identified using a three-color immunofluorescence method, including FITC-conjugated anti-cytokeratin (CK) and anti-EpCAM markers for epithelial cells, a PE-conjugated anti-CD45 marker for white blood cells (WBCs), and DAPI for nuclear staining. Cells that were CK^+^ or EpCAM^+^, CD45^-^, DAPI^+^, and morphologically intact were identified as CTCs, while cells that exhibited high CD45 expression levels were identified as WBCs. Fluorescently stained cells were automatically scanned on a slide using a BioView workstation (BioView, Inc.) for the FAST disc.

### CTC isolation and analysis using CellSearch

CTC enumeration, immunofluorescent staining, and detection by the CellSearch epithelial cell test (Menarini Silicon Biosystems, Inc.) were performed using the CellSearch™ protocol (www.cellsearchctc.com) at the Brigham and Women's Hospital (BWH), Boston, MA (The BWH IRB number is 2016P001708). A FAST disc operation system was shipped to BWH and used for a direct comparison with CellSearch. Two blood samples were collected from 17 enrolled patients with cancer into two types of tubes: 7.5 mL of whole blood in CellSave tubes (Menarini Silicon Biosystems, Inc.) for CellSearch and 3 mL of whole blood in EDTA tubes for FAST disc detection. The enumeration of CTCs was performed using the recommended protocols for each method, as described previously 25, 29, 30.

### DNA mutation analysis

To detect mutations in isolated CTCs, genomic DNA was extracted using the QIAamp DNA blood mini kit (Qiagen). For the detection of L858R, 19del and T790M mutations in gDNA, PCR was performed using the following cycling conditions on a Master cycler pro S instrument (Eppendorf): 95 °C for 10 min, followed by 40 cycles of 95 °C for 30 s and 58 °C for 1 min, and 98 °C for 10min, then held at 4 °C. The PCR mixture (20 µL) contained 10 µL of ddPCR supermix for probes, no UTP (Bio-Rad), 1 µL of 20× primer/probe assay for mutant and wild type (Bio-Rad), and 8 µL of DNA. The copy number of mutants was quantified using QuantaSoft software (Bio-Rad).

### Statistical analysis of CTC enumeration data

Overall survival (OS) and progression-free survival (PFS) were chosen as the endpoints to evaluate the prognostic value of CTC counts. The survival time was estimated using the Kaplan-Meier method, and the difference in survival between groups was assessed using a log-rank test. The SPSS 22.0 for Windows software was used for statistical analyses. To confirm the correlation between the CTC count change ratio; 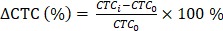 where CTC*_i_* is the CTC count at time point *i* and CTC_0_ is the baseline CTC count and imaging response results, a two-tailed Student's *t*-test with a 95% confidence interval was performed using Origin 2015 (OriginLab Corp.). *P*-values < 0.05 were considered statistically significant.

### Single-cell isolation and multiple gene expression analysis

Single cells were isolated using Kuiqpick™ (NeuroInDx). Cells captured on the membrane were stained for CD45, EpCAM, and DAPI to identify target cells. Single-cell cDNA was prepared using the Single Cell-to-Ct kit (Life Technologies), and a specific target was preamplified for gene expression analysis. qRT-PCR was performed using a BioMarkHD real-time PCR system and the software (Fluidigm). For analysis, quality thresholds of 0.65 and Ct ≤ 30 were considered.

Undetected genes were assigned a Ct-value of 999, which was imputed as the highest Ct-value observed for a given gene plus a value of 1 to provide balanced weights to missing data. All imputed Ct-values were converted to Z-scores to provide the same weights 31. To assign equal weights to all measured mRNAs, the gene expression values were mean-centered and Z score-transformed by dividing the mean-centered expression value by the standard deviation. We conducted unsupervised hierarchical clustering and t-distributed stochastic neighbor embedding (t-SNE) to explore associations among sample groups. Unsupervised hierarchical clustering and its heatmap visualization were performed using the *heatmap.2* function of the *gplots* package, and t-SNE analysis was performed using the *Rtsne* package in R (version 3.4.0).

Correlation matrix plots were constructed using the *corrplot* function in R. Correlation matrices were computed separately for patient-derived CTCs, cancer cell lines, and WBCs, with Spearman's rank correlations. Associated *P*-values were computed using the *cor.mtest* function in R. The Bonferroni correction of *P*-values was performed to adjust for multiple testing in the rank correlation matrix.

### CTC classification

Based on the gene expression profiling data for individual cells, we calculated their epithelial (E) or mesenchymal (M) scores. Epithelial markers such as EpCAM and CK markers (KRT7, KRT18, and KRT19) were selected to measure scores related to epithelial characteristics of CTCs. The mesenchymal markers vimentin and CD44 were selected to calculate M-scores of CTCs. The mRNA expression values of the target markers, normalized to those of *GAPDH* using the 2^-ΔCt^ method, were added up for each E/M group, and the relative percentage was used to classify CTCs according to their epithelial-to-mesenchymal transition (EMT) characteristics.

## Results

### Label-free isolation of CTCs from whole blood using FAST disc

**Figure 1:**
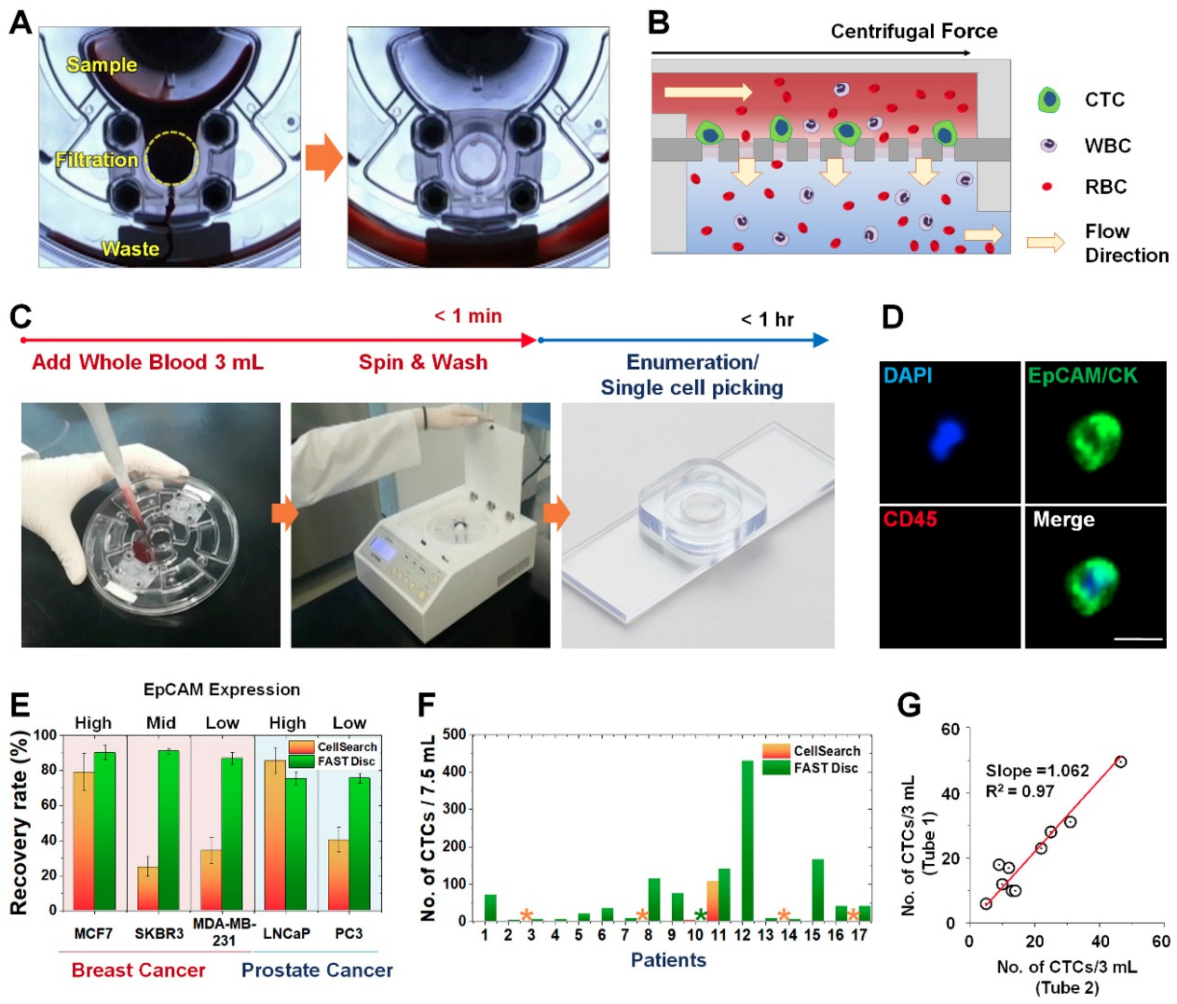
** Workflow, mechanism, and performance of FAST disc. (A)** Visualization of the FAST disc before and after the CTC enumeration process. **(B)** The centrifugal force pushes the liquid in a tangential direction to the flow filtering through the membrane. **(C)** Size-based isolation of CTCs from whole blood (3 mL) can be completed within 1 min, all in one disc. After washing and staining for enumeration, the filter can be removed and mounted on a slide glass for downstream analysis. **(D)** Representative images of CTCs (DAPI^+^, EpCAM/CK^+^, CD45^-^) isolated from the blood of cancer patients. **(E)** Performance comparison between the CellSearch system and FAST disc using whole blood spiked with cancer cells (<100). Five cell lines with various EpCAM expression levels were used to quantify the recovery rates. **(F)** CTCs enumerated by the CellSearch system and FAST disc from the blood samples of 17 patients with different cancer types. **(G)** Reproducibility test using blood samples from one patient collected in two independent blood collection tubes showing similar CTC counts.

A FAST disc is a centrifugal microfluidic device, which enables clog-free, label-free CTC isolation from whole blood 25 (**Figure 1A**). In a FAST disc, the fluid flow by centrifugal force and that by filtration through the membrane are in perpendicular directions, similar to the tangential-flow filtration, which minimizes clogging (**Figure 1B**). The operation of the FAST disc is very simple and fast: 1) add 3 mL of whole blood to the sample-loading chamber; 2) spin the disc using a tabletop-sized, portable spinning machine; and 3) add 1 mL of washing buffer and spin. The total process of the isolation of CTCs from 3 mL of whole blood could be finished within 1 minute (**Figure 1C** and**Movie S1**). The CTC enumeration was performed using the conventional criteria for CTC identification, EpCAM/CK^+^ and CD45^-^, with immunofluorescence staining performed on the disc (**Figure 1D and Figure S1**).

Compared with a commercially available product, the CellSearch system (Menarini Silicon Biosystems, Inc.), the FAST disc showed a high capture efficiency, irrespective of the EpCAM expression levels in the cell lines, while CellSearch showed a high capture efficiency only for cell lines with high EpCAM expression (**Figure 1E**). Further, clinical tests using blood samples from 17 patients with various cancer types demonstrated that size-selective CTC isolation using the FAST disc outperformed the immunoaffinity-based enumeration by CellSearch; the detection rates were 94.1% and 11.8%, respectively. In addition, the failure rates were much lower for the FAST disc than for CellSearch, 5.9% and 23.5%, respectively (**Figure 1F**). Measurement of CTCs in two blood samples collected from the same patient confirmed a good reproducibility of the FAST disc platform, which is a prerequisite for continuous monitoring of the disease status** (Figure 1G).**

### Clinical characteristics of the prospective cohort of patients with NSCLC receiving EGFR-TKIs treatment

A total of 340 blood samples were obtained from 40 patients diagnosed with NSCLC with *EGFR* mutations at different treatment time points for CTC isolation using the FAST disc platform; a summary of the patients' baseline characteristics is shown in **Table 1**. At the time of analysis, 22 of the 40 patients (55.0%) experienced disease progression, and 12 patients (30.0%) died or were lost to follow-up, resulting in a median PFS of 13.1 months (95% CI: 11.7‒16.3 months) and OS of 19.0 months (95% CI: 15.6‒20.2 months). The average follow-up time for the 28 patients who were still alive was 19.6 months (range: 9.8‒31.3 months).

### Baseline CTC counts and survival

The median CTC_0_ value among 38 tested patients was 37 CTCs/7.5 mL. With the cutoff value of 66 CTCs/7.5 mL, which minimized the p-value from the log-rank test, eight patients (21.1%) showed CTC counts higher than the cutoff (CTC^High^ group). Thirty patients (78.9%) showed CTC counts lower than the cutoff (CTC^Low^ group). As shown in **Figure S2A**, the CTC^Low^ group had a relatively longer OS than did the CTC^High^ group, but the difference was not statistically significant. Thus, no correlation was observed between CTC_0_ and OS. One possible reason is that the efficacy of the first-line therapy, rather than CTC_0_ itself, might be more relevant to OS of the prospective cohort of patients tested in this study (**Figure S2B-C**).

### Serial monitoring of CTC counts

**Figure 2:**
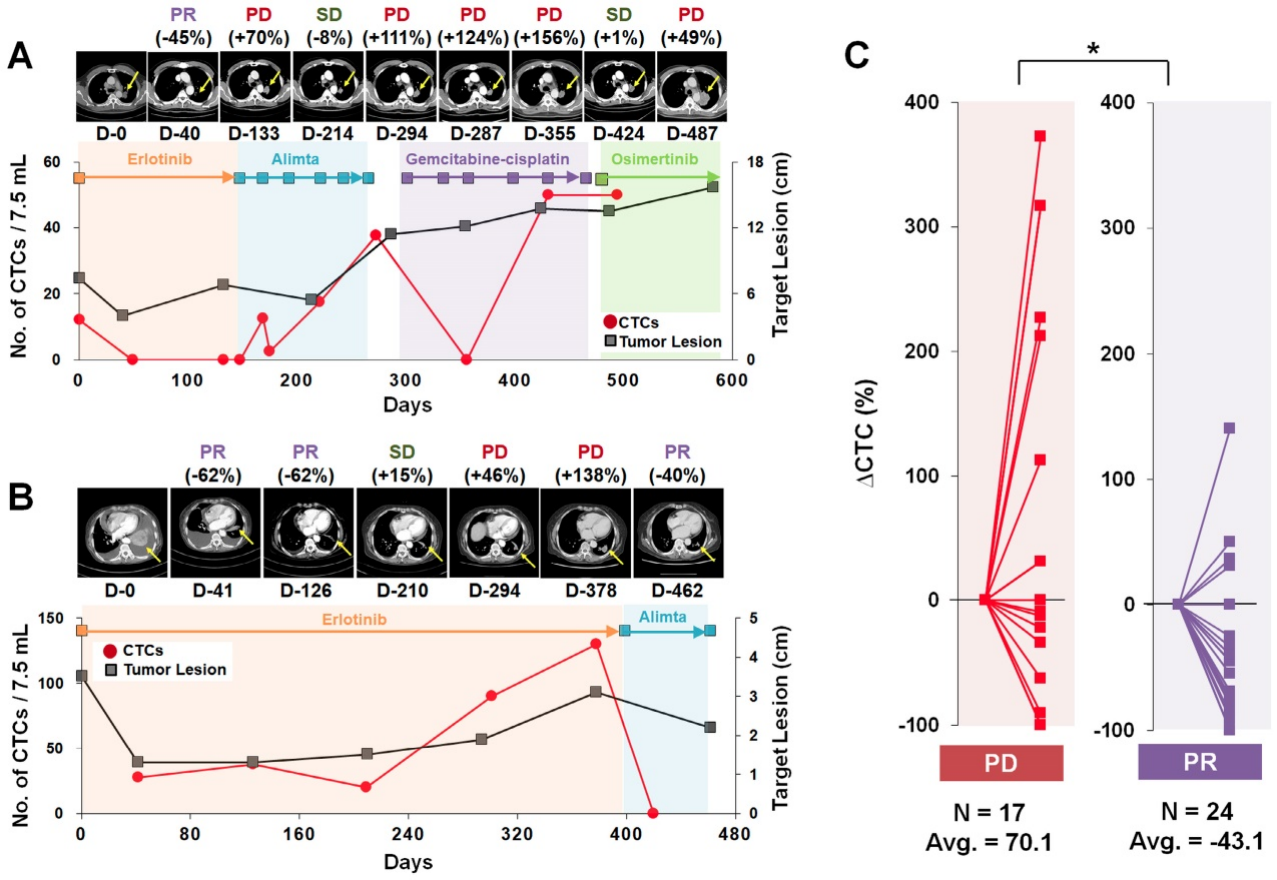
** Representative examples of CTC counts and CT images obtained during the course of treatment and their correlations. (A)** Tumor size and CTC counts show an increase in the tumor burden during 582 days of various EGFR-TKI treatments for patient LP2. **(B)** Patient LP11 continued to receive erlotinib initially. The CTC counts from the blood draw at 301 and 378 days were significantly increased with PD determined from CT images obtained on days 294 and 378. After the treatment was changed to alimta, both the CTC counts and tumor burden decreased dramatically and PR was determined at day 462. **(C)** Relationships with the change rate of CTC counts, △CTC (%); CTC counts change normalized by the baseline CTC, and CT scan image response results from patients with a 19 del mutation who enrolled in the follow-up monitoring study for more than 19 months. The CTC count change rate is sorted according to the imaging response results (PD or PR). The PD group showed a significantly greater CTC count change rate than the PR group (P = 0.011).

We next focused on the trends of CTC counts to evaluate the correlation with patients' disease burden and treatment response, as shown in **Figure 2.** A cohort of 11 patients who had the 19del mutation and were enrolled for more than 19 months was used to evaluate the correlation of CTC counts with the treatment response. The RECIST criteria (version 1.1) were used to assess the treatment response via computed tomography (CT) scans acquired every two or three cycles, and responses were classified as a complete response (CR), partial response (PR), stable disease (SD), or progressive disease (PD) 32. When we compared the change rate of the CTC counts as a function of the imaging response results, the PD group (average 70.1% increase, N=17) showed a significantly higher CTC count change rate than did the PR group (average 43.1% decrease, N=24).

### Detection of *EGFR* mutations in tumor tissue and CTCs isolated using the FAST disc

We evaluated the concordance rate in detecting mutations in tumor tissue and CTCs isolated from blood samples. In 15 samples from 13 patients, the mutations identified in CTCs were 100% consistent with those found in the matching tumor tissues. Interestingly, the *EGFR* T790M mutation was detected in both relapsed tissue and CTCs isolated from blood samples of two patients, LP2 and LP49, who did not show this mutation during their first biopsy using both tumor tissue and blood samples, as summarized in **Table 2**.

### Single-cell RNA expression analysis of patient-derived CTCs

**Figure 3:**
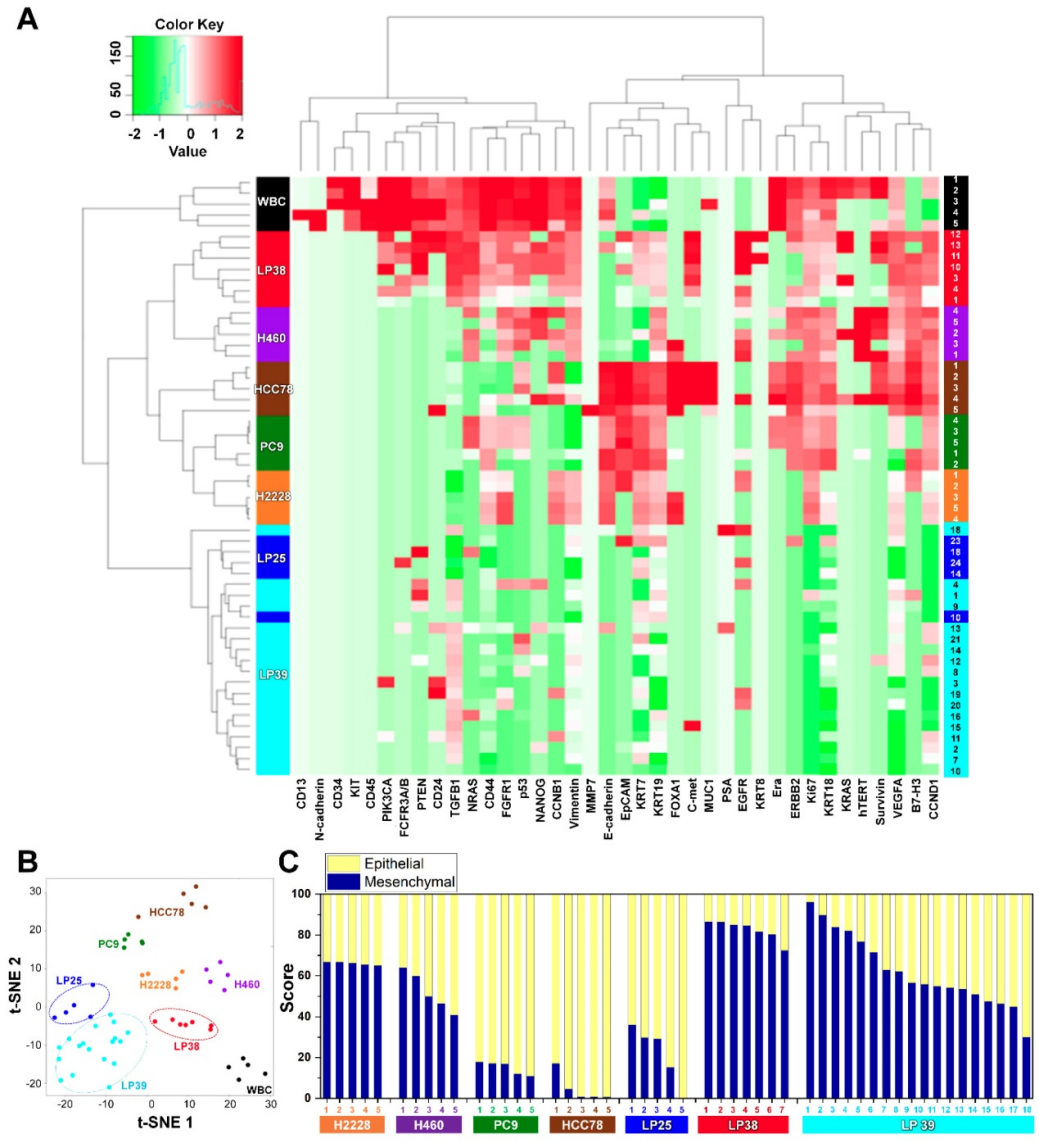
** Gene expression profiles of single CTCs isolated from three patients with lung cancer and from lung cancer cell lines. (A)** Heat map for the hierarchical clustering of the differentially expressed genes of single cells from four different NSCLC cell lines [H2228 (orange), H460 (purple), HCC78 (brown), and PC9 (green)], WBCs (black), and CTCs from three patients [LP25 (red), LP38 (blue), and LP39 (cyan)]. Each row represents a single-cell sample. The scale bar indicates the Z-scores of gene expression values, with highly expressed genes depicted in red and low-expressed genes depicted in green. **(B)** Two-dimensional t-SNE analysis based on hierarchical clustering. Cells clearly grouped according to the sample source. **(C)** Gene expression data of individual cells were further characterized to show an epithelial or mesenchymal cell signature. Intra- and intercellular heterogeneity is demonstrated using four different NSCLC cell lines, and profiling of individual CTCs isolated from three patients with NSCLC patients before chemotherapy. Patients LP25 and LP39 carried an 19del mutation, and patient LP38 had the L861Q mutation.

To characterize the tumor heterogeneity, we applied a single-cell manipulation technique to isolate patient-derived live CTCs. After blood filtration and immunostaining for CD45 without a fixation step, live CTCs captured on the membrane were collected, and individual cells without a CD45 signal were subjected to single-cell RNA expression analysis **(Figure S3** and** Figure 3)**.

We assessed mRNA expression in individual cells isolated from the four lung cancer cell lines, H2228, H460, HCC78, and PC9 (**Figure S4**), and from the blood samples withdrawn from three patients with NSCLC, LP25, LP38, and LP39, before starting their EGFR-TKIs therapy, using WBCs as a control (**Figure S5**). Differences in the gene expression were confirmed between the cell lines, and CD45 was only detected in WBCs. The list of genes used in the single-cell qRT-PCR experiment is presented in **Table S1**. To explore associations among different cell groups, the data were analyzed using unsupervised hierarchical clustering (**Figure 3A**). It is obvious that individual cells show heterogeneous gene expression profiles, while single cells from the same group are clustered together, which implies a higher degree of interpatient heterogeneity of CTCs. In addition, the t-SNE analysis, shown in **Figure 3B**, clearly presents the inter-cell line and interpatient heterogeneity of CTCs. A correlation matrix plot showing Spearman's correlation coefficient among 30 baseline CTCs isolated from three patients, five WBCs, and 20 cells from the four different NSCLC cell lines (H2228, H460, HCC78, and PC9) is shown in **Figure S6**.

We further characterized an epithelial or mesenchymal signature of individual CTCs, based on previous reports 33-35. Epithelial CTCs showed high levels of the epithelial marker EpCAM and CK markers, KRT7, KRT18, and KRT19, and low levels of mesenchymal markers, vimentin and CD44, whereas mesenchymal CTCs expressed the opposite trend. Individual cells from the four different NSCLC cell lines and three different patients with NSCLC showed different E/M hybrid signatures **(Figure 3C** and **Figure S7A)**. Based on the classification criteria, the H2228 cell line had an M-score of 66.0% ± 0.7%, which was consistent with its reported EMT properties 36. The HCC78 and PC9 cell lines had the highest expression ratios of the epithelial markers, 95.2% ± 6.3% and 85.0% ± 2.9%, respectively. The H460 cell line had an intermediate expression ratio, with an M-score of 52.2% ± 8.5%. Despite their heterogeneity, single cells from the same cell lines were clustered together when we plotted the gene expression of the mesenchymal markers, vimentin and CD44, against that of the epithelial markers, EpCAM, KRT7, KRT18, and KRT19 (**Figure S8**).

Further, distinct differences in gene expression patterns among single CTCs from the three patients were clearly more significant than the heterogeneity found among individual CTCs isolated from each patient. Five CTCs from patient LP25 mainly exhibited the epithelial signature, whereas seven CTCs from patient LP38 were mesenchymal. On the other hand, 18 CTCs from patient LP39 were more heterogeneous, with the intermediate expression of mesenchymal markers **(Figure 3C** and**Figure S7A)**. High expression of EMT markers is known to be characteristic of more aggressive CTCs 37. Although the number of samples was very limited in this exploratory study, patient LP38, whose CTCs showed the highest mesenchymal gene expression, had the shortest survival, whereas patient LP25, whose CTCs mainly showed epithelial characteristics, had the longest survival. The survival periods of patients LP25, LP38, and LP39 were 624, 212, and 468 days, respectively.

### Serial monitoring of single-cell RNA expression in CTCs from patients with NSCLC during EGFR-TKI treatment

**Figure 4:**
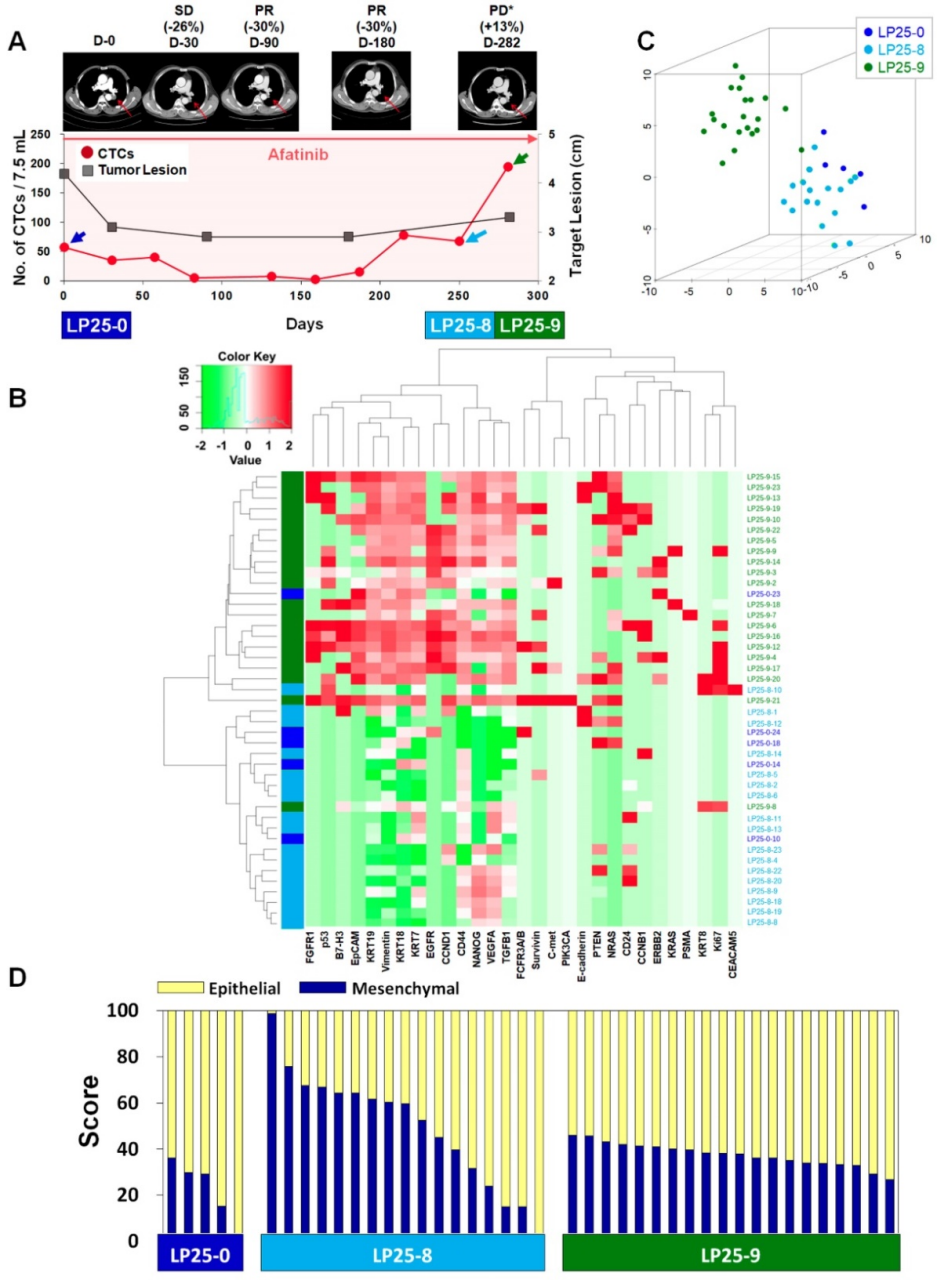
** Longitudinal study of LP25 with CTC counts, individual CTC profile, and CT image response during TKI therapy. (A)** Correlation between the number of CTCs and tumor size from CT images in patient LP25 with EGFR-mutant NSCLC during the course of TKI therapy. **(B)** Heatmap of the hierarchical clustering of gene expression profiles of single CTCs. **(C)** Three-dimensional t-SNE plots for CTCs isolated at three time points during the therapy of patient LP25. Despite the inter-sample heterogeneity of CTCs, cells clustered according to the same time point during the treatment. **(D)** EMT characterization of single CTCs from patient LP25 during TKI treatment.

**Figure 5:**
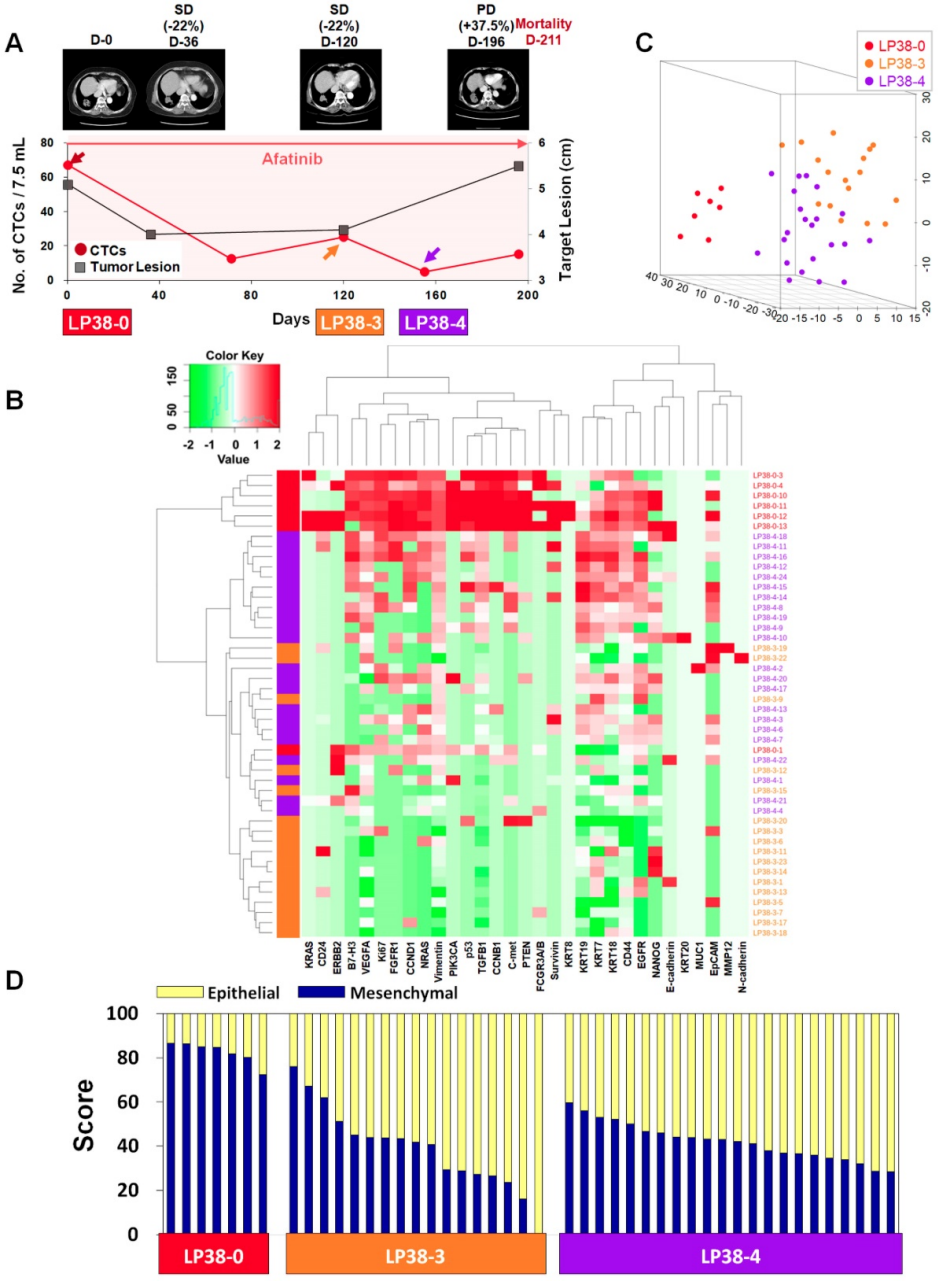
** Longitudinal study of patient LP38 with CTC counts, individual CTC profile, and CT image response during TKI therapy. (A)** Correlation between the number of CTCs and tumor size from CT images in patient LP38 with EGFR-mutant NSCLC during the course of TKI therapy. **(B)** Heatmap of the hierarchical clustering of gene expression profiles of single CTCs. **(C)** Three-dimensional t-SNE plots for CTCs isolated at three time points during the therapy of patient LP38. Despite the inter-sample heterogeneity of CTCs, the cells clustered according to the same time point during the treatment. **(D)** EMT characterization of single CTCs from patient LP38 during TKI treatment.

For treatment response monitoring using CTCs, we performed a single-cell-level mRNA gene expression analysis using individual CTCs isolated at three time points during chemotherapy, in addition to regular measurements of CTC counts and CT image responses. For this proof of concept study, we enrolled two prospective patients, LP25 (**Figure 4**) and LP38 (**Figure 5**), during the period of EGFR-TKI (afatinib) treatment.

In the case of patient LP25, the initial treatment response was relatively good, and the number of CTCs/7.5 mL of blood decreased from 57.5 at baseline to 2.5 after 159 days of treatment, which was consistent with the CT scan-based SD and PR statuses after 30, 90, and 180 days of treatment, as shown in **Figure 4A**. However, the CTC counts increased to 15.0, 67.5, and 195.0 per 7.5 mL after 187, 250, and 281 days of treatment, which was in good agreement with radiographic data, indicating PD, at 282 days of treatment.

In contrast, patient LP38 showed 67.5 CTCs/7.5 mL at baseline, but the counts decreased to 12.5, 25.0, 5.0, and 15.0 at 71, 120, 155, and 196 days of treatment, respectively. Although the patient initially showed an SD response, until 120 days of treatment, the tumor size increased again, scoring PD, at 196 days, and the patient died 211 days after entering the study.

Although the dynamic changes in the numbers of CTCs were overall consistent with the CT data and clinical outcomes, the CTC counts were not sufficient to predict clinical outcomes. We hypothesized that, in addition to the total number of CTCs, the heterogeneity of individual CTCs and their characteristics might affect the patient outcome. Therefore, we tracked the gene expression characteristics of individual CTCs during the time course of TKI therapy.

For patient LP25, the single-cell-level gene expression profiling was performed using 5, 17, and 20 CTCs isolated before (LP25-0), after 250 days (LP25-8), and after 281 days (LP25-9) of treatment, respectively **(Figure 4** and**Figure S9**). From patient LP38, 7, 17, and 22 CTCs were isolated before (LP38-0), after 120 days (LP38-3), and after 155 days (LP38-4) of treatment, respectively **(Figure 5** and**Figure S10**). The numbers of CTCs used for the gene expression analysis **(Figure 4B-D** and** Figure 5B-D)** did not reflect the total numbers of CTCs, which were more carefully enumerated using an additional blood sample, as shown in **Figure 4A** and **Figure 5A**. As shown in the 3D t-SNE plots in **Figure 4C** and** Figure 5C**, each individual CTC showed a different gene expression pattern in hierarchical clustering, but CTCs from the same time point were well clustered together for both patients. In addition, the gene expression profiles of CTCs isolated at different time points were distinctly different, which was also demonstrated by the correlation matrix analysis (**Figure S11** and **Figure S12**).

We further characterized the EMT signatures of individual CTCs isolated during the time course of therapy. As shown in **Figure 4A**, patient LP25 showed SD and PR based on the CT scan results until 180 days, but the status changed to PD because of a new brain lesion at 282 days (LP25-9). Interestingly, the LP25-9 CTCs showed much higher expression of many more genes than did the LP25-0 or LP25-8 CTCs. When we classified individual CTCs based on the epithelial or mesenchymal gene expression signature, the heterogeneity of individual CTCs, as well as the dynamic change of the M-score, was obvious, as shown in **Figure 4D**. The M-score of CTCs at baseline (LP25-0) was relatively low (22.06% ± 12.96%) but increased, with a large standard deviation (49.53% ± 24.80%), for CTCs isolated after 250 days (LP25-8), which was approximately 1 month before PD was scored, with a new lesion in the brain. Interestingly, CTCs obtained on day 282, which was when a CT scan was taken, and PD was scored (LP25-9), showed a slightly decreased M-score, with a much lower intra-sample heterogeneity (37.51% ± 4.96%; **Figure S7B)**.

Patient LP38 had the highest expression of many genes in baseline CTCs (LP38-0), which somewhat decreased in CTCs isolated after 120 days of treatment (LP38-3), but with a significantly enhanced heterogeneity (**Figure 5D** and**Figure S7C**). Likewise, the tumor size was the largest at LP38-0 and decreased by 22% as the target lesion was scored SD (LP38-3). However, CTCs isolated after 155 days of treatment (LP38-4) again showed increased gene expression signals, which was consistent with the future clinical outcome and a PD score after 196 days of treatment. The EMT signature of individual CTCs was the strongest (82.39% ± 4.61%) at baseline (LP38-0), which dramatically decreased, with a high heterogeneity (39.24% ± 18.30%), in the LP38-3 CTCs and then remained at a similar level but with a lower heterogeneity (42.09% ± 8.31%) in the LP38-4 CTCs. Unfortunately, the amount of the blood sample obtained on day 196 was not sufficient for single-cell analysis, and the patient expired after 15 days.

## Discussion

The availability of more facile methods for measuring the disease progression and identifying predictive biomarkers of therapeutic responses in patients with NSCLC would significantly contribute to clinical outcomes of patients. Recent advances in liquid biopsy technologies have enabled less invasive access to tumor materials, highlighting the prognostic value of CTCs in disease management 38-42. However, frequent monitoring of CTCs has been limited by inconsistency in the enrichment yield of the extremely rare and heterogeneous cells, as well as by high costs and complexity of experimental protocols. Our study of prospectively collected blood samples from patients with NSCLC before and during EGFR-TKI therapy showed that longitudinal analysis of CTCs enumerated using a label-free isolation approach might provide early access to monitoring disease responses to targeted therapy.

Many studies have demonstrated that pretreatment CTC counts are highly relevant to PFS or OS of patients with advanced NSCLC 11, 12. However, there have also been conflicting reports showing no statistical significance in the survival outcome depending on baseline CTC counts 17, 18. A likely explanation for this discrepancy is the lack of standard enumeration methods for extremely rare and heterogeneous CTCs, as well as differences in the type of patients and the choice of treatment options. In our study, CTCs were detected in the majority of patients (85%), but the baseline CTC counts did not show an association with PFS or OS. On the other hand, dynamic changes in CTC counts were associated with CT scan-based tumor responses, suggesting a potential utility for early identification of responses to therapies. Although our data are consistent with those of a previous report 43, the number of patients was limited, and a further study, with a larger number of prospective patients in a setting with other targeted therapies or chemotherapy, is required to validate these findings.

Frequent monitoring of tumor mutations in patients with lung cancer is an urgent need to provide a clinically important guidance for disease management 44-47. For most patients with NSCLC, who harbor *EGFR* mutations, the treatment with an EGFR-TKIs therapy is beneficial. However, the T790M point mutation may arise soon, indicating the development of resistance to EGFR-TKI and the necessity to change the drug to third-generation EGFR-TKIs. Tissue biopsy is highly invasive, which prevents frequent monitoring of the development of mutations, and may not provide a representative mutation profile for patients with heterogeneous tumors at multiple sites. In this study, we demonstrated 100% concordance in the detection of *EGFR* mutations in tumor tissue and CTCs isolated from a total of 15 samples collected from 13 patients, including two cases of the emergence of the T790M mutation. These results present a promising outlook on the potential of routine analysis of CTCs to provide clinically relevant information for the timely selection of personalized therapies.

Other molecular information, such as gene expression data, can also be obtained by downstream analysis of a single CTC. Direct analysis of bulk cells may generate background signals from WBCs and other blood cells 48. Furthermore, bulk cell-level analysis, based on signal averaging, leads to losing individual characteristics of heterogeneous CTCs, which are related to tumor biology and metastatic potential 49. The importance of CTC characterization at a single-cell level has been highlighted by profiling multigene expression in individual CTCs in a heterogeneous population 19, 46, classifying CTC subpopulations 31, and studying an association of CTCs with the clinical outcome or tumor biology 50. Herein, by using a label-free, high-throughput, clinical setting-friendly method for CTC isolation, based on the previously reported tabletop-sized FAST disc system 25, we were able to perform a longitudinal analysis of single-cell gene expression in CTCs isolated before and during targeted therapy. Our findings from single-cell gene expression analysis of four different lung cancer cell lines and three different patients indicated that the inter-heterogeneity among different cell lines or patients was much higher than the intra-heterogeneity within each group of CTCs.

EMT plays a key role in tumor progression and metastasis. EMT is associated with the loss of the epithelial phenotype, and increased invasion, migration, and cell proliferation are associated with the increased expression of mesenchymal markers. A number of studies have suggested that EMT may be associated with the resistance to EGFR-TKIs in patients with NSCLC 51, 52. It was also suggested, based on liquid biopsy applications, that CTCs expressing EMT-related genes were associated with the disease progression and poor therapeutic responses in patients with breast 33, gastric 34, pancreatic 31, and lung 53 cancer. Gene expression in single cells has been studied to evaluate the heterogeneity of individual CTCs. Thus, Lin et al. 35 characterized single CTCs and demonstrated their inter- and intra- patient heterogeneity in four patients with pancreatic cancer. Ting et al. 54 conducted single-cell RNA sequencing of pancreatic CTCs and identified three distinct populations, with the data suggesting multiple pathways in the metastatic cascade. However, molecular analysis of single CTCs remains challenging because currently available platforms are expensive and not robust enough to be translated into the clinic. Consequently, there have been no reports on longitudinal monitoring of therapeutic responses via profiling of the EMT signature in individual CTCs based on a single-cell gene expression analysis.

EMT is crucial for cancer cells to acquire invasive and migratory abilities, eventually driving tumor metastasis. Recent studies have reported that EMT is not a step function, in which the change occurs from the E to the M state and that cells rather exhibit a hybrid E/M phenotype with a mix of E/M traits, which may be highly correlated with patient outcomes 52, 55, 56. One important result of this study was that the E/M signature scores obtained by multigene expression analysis of single cells revealed substantial heterogeneity among individual cells and among cell lines. Despite their relatively small intra-heterogeneity, the EMT scores of individual cells from four different NSCLC cell lines could predict the known characteristics of the specific cell types, which were categorized as mainly mesenchymal or epithelial, or a hybrid state, showing both traits 52, 57, 58. Furthermore, analysis of EMT scores of individual pretreatment CTCs isolated from three patients with NSCLC indicated that the patients who showed relatively poor drug responses had CTCs with high M-scores, while the patient who showed a relatively good response to EGFR-TKI therapy had CTCs with high E-scores. Despite the heterogeneity in multigene expression and EMT scores among CTCs isolated from the patients, CTCs from the same source were clustered together, suggesting that the interpatient heterogeneity was higher than the intra-patient one.

We also monitored how multigene expression and EMT signatures evolved during EGFR-TKI therapy. Based on the data obtained using a limited number of CTCs isolated from two patients, the EMT scores of pretreatment CTCs were better associated with the drug response than were those of CTCs obtained during EGFR-TKI therapy. Even though the EMT scores of pretreatment CTCs were distinctly different (predominantly E or M), the EMT signatures of individual CTCs became more heterogeneous and then changed to a relatively uniform E/M hybrid signature during EGFR-TKI treatment. However, the meaning of the change in multigene expression profiles should be further validated in relation to drug responses in future studies, involving large cohorts of patients.

Although we only performed gene expression analysis using the Fluidigm technology, CTCs isolated without antibody selection bias can be applied for other types of molecular analysis at a single-cell level to unveil the tumor heterogeneity during disease progression upon personalized therapy. Despite the significantly reduced turnaround time and minimized manual operation steps, the CTC counting and picking process still require manual operation, which need to be automated for broader usage in clinical settings. A potentially important application of our technology is the isolation of CTC clusters, which are emerging as important players in metastasis. Though we could frequently observe CTC clusters from patients with other types of cancer, CTC clusters were rarely observed from patients with NSCLC (**Figure S1B**). In recent studies on inertial microfluidics-based CTC isolation using blood samples from patients with NSCLC, Zhou et al. did not observe CTC clusters 23, while Zeinali et al. detected CTC clusters from the majority of patients 24. We believe that this discrepancy might be due to the different cohort of patients with relatively limited number of clinical samples.

In conclusion, we demonstrated that the label-free and clinical setting-friendly FAST disc platform enabled rapid enumeration of CTCs and their unbiased molecular analysis at a single-cell level. Our approach can be readily applied in future clinical studies for early detection of actionable mutations for therapy selection and disease monitoring. Our findings from an exploratory, small-scale, prospective cohort study of *EGFR*-mutant patients with NSCLC support the importance of single-cell-level molecular analysis, including an EMT signature, to investigate the dynamics of disease progression and predict the drug response. We anticipate that the FAST disc system can become a clinical research tool to achieve comprehensive molecular profiling and assess the heterogeneity of CTCs from patients undergoing targeted therapy and to monitor the association with clinical outcomes. Overall, high-throughput, label-free isolation of CTCs from whole blood using the FAST disc platform may facilitate minimally invasive characterization and frequent monitoring of tumor progression for timely selection of a personalized medicine.

## Supplementary Material

Supplementary figures and table.

Supplementary movie.

## Figures and Tables

**Table 1:** Patients demographics and clinical characteristics

Characteristics	No. (%)
**Age at baseline,** years	
Median	63
range	34-83
**Sex**	
Male	15 (37.5)
Female	25 (62.5)
**M stage at diagnosis**	
M0	3 (7.5)
M1a	6 (15)
M1b	31 (77.5)
**EGFR mutation**	
19del	19 (47.5)
L858R	16 (40)
Others	5 (12.5)
**EGFR-TKI received**	
Gefitinib	2 (5)
Erlotinib	9 (22.5)
Afatinib	29 (72.5)

**Table 2:** ** Detection of EGFR mutation from CTCs.** The mutation detection results from CTCs isolated from total 13 patients were in 100% concordance with the corresponding results obtained from tissue biopsy sample. Notably, T790M, the acquired resistance EGFR mutation, detected at relapse (AR) of samples, but not before the treatment (BT), from two patients (LP49 and LP2) both from noninvasive blood-based CTC analysis as well as tumor biopsy.

**Patient**	**Sex**	**Age**	**Stage**	**L858R**		**T790M**	
				**Tissue**	**CTC**	**Tissue**	**CTC**
LP1	F	52	IV	+	+	-	-
LP5	M	49	IV	+	+	-	-
LP6	M	58	IV	+	+	-	-
LP8	F	76	IV	+	+	-	-
LP10	M	63	IV	+	+	-	-
LP12	F	90	IV	+	+	-	-
LP13	F	53	IV	+	+	-	-
LP46	F	67	IV	+	+	-	-
LP47	F	83	IV	+	+	-	-
LP49 (BT)	F	65	IIIA	+	+	-	-
LP49 (AR)	F	65	IIIA	+	+	+	+
**Patient**	**Sex**	**Age**	**Stage**	**19 del**		**T790M**	
				**Tissue**	**CTC**	**Tissue**	**CTC**
LP2 (BT)	M	53	IV	+	+	-	-
LP2 (AR)	M	53	IV	+	+	+	+
LP25	M	74	IV	+	+	-	-
LP43	F	59	IV	+	+	-	-
				***100%***		***100%***	
