# Supplementary material for: A lab-on-a-disc platform enables serial monitoring of individual CTCs associated with tumor progression during EGFR-targeted therapy for patients with NSCLC
Source: Theranostics. 2020 Apr 6;10(12):5181–94. doi: 10.7150/thno.44693 (PMC7196290; doi:10.7150/thno.44693)
Supplement: Supplementary file 1 — Supplementary figures and table. [file thnov10p5181s1.pdf]

Supplementary Information

for

A lab-on-a-disc platform enables serial monitoring of individual CTCs associated with tumor progression during EGFR-targeted therapy for patients with NSCLC

Minji Lim[1, 2 †], Juhee Park[1 †], Alarice C. Lowe[3 ‡], Hyoung-oh Jeong[2], Semin Lee[2], Hee Chul Park[4], Kyusang Lee[4], Gwang Ha Kim[5], Mi-Hyun Kim[5]* and Yoon-Kyoung Cho[1, 2]*

[1]Center for Soft and Living Matter, Institute for Basic Science (IBS), Ulsan 44919, Republic of Korea

[2]Department of Biomedical Engineering, School of Life Sciences, Ulsan National Institute of Science and Technology (UNIST), Ulsan 44919, Republic of Korea

[3]Department of Pathology, Stanford University, Stanford, CA, 94305, USA

[4]Clinomics Inc., Ulsan 44919, Republic of Korea

[5]Department of Internal Medicine, Pusan National University School of Medicine and Biomedical Research Institute, Pusan National University Hospital, 179, Gudeok-ro, Seo-Gu, Busan 49241, Republic of Korea

[‡]Alarice C. Lowe's previous affiliation: Brigham and Women's Hospital, Harvard Medical School, Boston, MA, USA

[†]These authors contributed equally to this work.

*Corresponding author: Mi-Hyun Kim, Department of Internal Medicine, Pusan National University School of Medicine and Biomedical Research Institute, Pusan National University Hospital, 179, Gudeok-ro, Seo-Gu, Busan 49241, Republic of Korea. E-mail: mihyunkim@pusan.ac.kr Phone: 82-51-240-7479; Fax: 82-240-8677; Yoon-Kyoung Cho, Center for Soft and Living Matter, Institute for Basic Science (IBS), Ulsan 44919, Republic of Korea; Department of Biomedical Engineering, School of Life Sciences, Ulsan National Institute of Science and Technology (UNIST), Ulsan 44919, Republic of Korea. ykcho@unist.ac.kr, Phone: 82-52-217-2511; Fax: 82-52-217-5509.

Supplementary Figure legends

Supplementary Figures

Figure S1. Fluorescence images of patient-driven CTCs. Representative examples of (A) patient-derived CTCs from two different NSCLC patients (Scale bar: 10 μm) and (B) CTC clusters from NSCLC patients (Scale bar: 8 μm). Merged images and three images from different fluorescent channels (DAPI, TRITC, and FITC) are shown. CTCs were defined as $DAPI^{+}/CD45^{-}/CK^{+}$ or $EpCAM^{+}$ cells.

Figure S2. Correlation between CTC counts and treatment responses on CT scans. (A) Kaplan-Meier curves for OS between two patient groups according to the cutoff baseline CTC count (66 CTCs/7.5 mL). (CTC^{Low}: n = 30, median OS: 18.04; CTC^{High}: n = 8, median OS: 15.46, log-rank p = 0.3501; samples from two patients had a clogging issue.) **(B)** Six of 40 patients (15%) did not respond well to their first choice of therapy and had to change the treatment option more than once, resulting in a significantly poorer PFS than that of patients who responded well to the first-choice drug. Kaplan-Meier curves for PFS between two patient groups according to the number of treated drugs. (N = 1: n = 34, median PFS: 25.70, N > 1: n = 6, median PFS: 9.65, log-rank p = 0.0039). **(C)** Kaplan-Meier curves for OS between the two patient groups according to the number of treated drugs, demonstrating no significant difference. (N = 1: n = 34, median OS: undefined, N > 1: n = 6, median OS: 28.50, log-rank p = 0.7518).

Figure S3. Procedures for single cell picking. (A) Schematic image of single cell picking. The membrane was separated from the disc and mounted onto the glass with the PDMS reservoir containing PBS. Using a capillary connected with a pump, a single cell was picked by the vacuum-assisted method. **(B)** Fluorescence images of a cell before and after picking from the membrane. $DAPI^{+}/CD45^{-}$ stained cells are candidates for analysis. Image set of DAPI, CD45, and EpCAM before picking showing the target cells, and the other DAPI image shows the same spot after picking. (Scale bar: 10 μm).

Figure S4. Single-cell real-time PCR using various lung cancer cell lines. Single-cell mRNA expression of four lung cancer cell lines, H2228, H460, PC9, and HCC78, determined using BiomarkHD qRT-PCR. After picking individual cells captured on the membrane by a cell-picker, RNA

was extracted and preamplified for single-cell mRNA expression analysis. Ct ≤ 30 with a Fluidigm real-time PCR analysis software quality threshold of 0.65 was considered for the analysis. Columns and rows show individual cells and the target genes assayed, respectively. N means no template control.

Figure S5. Single-cell real-time PCR using patient-driven CTCs from baseline samples and white blood cells. Single-cell mRNA expression of CTCs from three different patients at baseline (LP25, LP38, and LP39) determined using BiomarkHD qRT-PCR with five WBCs. Columns and rows show individual cells and the target genes assayed, respectively. N means no template control.

Figure S6. **Correlation matrix plots of correlations among 20 cells from four different cancer cell lines, five WBCs, and 30 patient-derived baseline CTCs isolated from three different patients.** Spearman correlation coefficients for sample characteristics. High and low similarity is indicated with blue and red color based on the scale bar, respectively. The circle size represents the magnitude of the correlation. P-values in this correlation analysis were derived using the Cor.mtest function in R. Bonferonni correction was applied to P-values to account for multiple testing in the rank correlation matrix.

Figure S7. Summary of mesenchymal scores. (A) Distribution of mesenchymal scores from each sample according to the EMT characterization among four NSCLC cell lines and three NSCLC patients. Mesenchymal scores of single CTCs from patients **(B)** LP25 and **(C)** LP38 during TKI treatment.

Figure S8. Correlation of marker expression among four NSCLC cell lines. (A) Vimentin expression of five cells from each cell line according to the expression of epithelial markers EpCAM, KRT7, 18, and 19, respectively. **(B)** CD44 expression of five cells from each cell line according to the expression of epithelial markers EpCAM, KRT7, 18, and 19, respectively.

Figure S9. Single-cell real-time PCR using CTCs from patient LP25 at various time points. Single-cell mRNA expression was determined using BiomarkHD qRT-PCR. Columns and rows show individual cells and the target genes assayed, respectively. N means no template control.

Figure S10. Single-cell real-time PCR using CTCs from patient LP38 at various time points. Single-cell mRNA expression was determined using BiomarkHD qRT-PCR. Columns and rows show individual cells and the target genes assayed, respectively. N means no template control.

Figure S11. Correlation matrix plots of CTCs before and after treatment in patient LP25. Spearman correlation coefficients for sample characteristics. High and low similarity is indicated with blue and red color based on the scale bar, respectively. The circle size represents the magnitude of the correlation.

Figure S12. Correlation matrix plots of CTCs before and after treatment in patient LP38. Spearman correlation coefficients for sample characteristics. High and low similarity is indicated with blue and red color based on the scale bar, respectively. The circle size represents the magnitude of the correlation.

Supplementary Table legends

Supplementary Table

Table S1. Genes used to profile single CTCs. 48.48 dynamic array microfluidic qRT-PCR chips (Fluidigm) used to confirm the expression of genes in all captured single cells.

Supplementary Movie legends

Supplementary Movie

Movie S1. Demonstration of the operation of a fluid-assisted separation technology (FAST) disc, which allows label-free, clog-free, and rapid (>3 mL/min) isolation of CTCs directly from unprocessed whole blood of patients using a tabletop-sized, portable spinning machine.

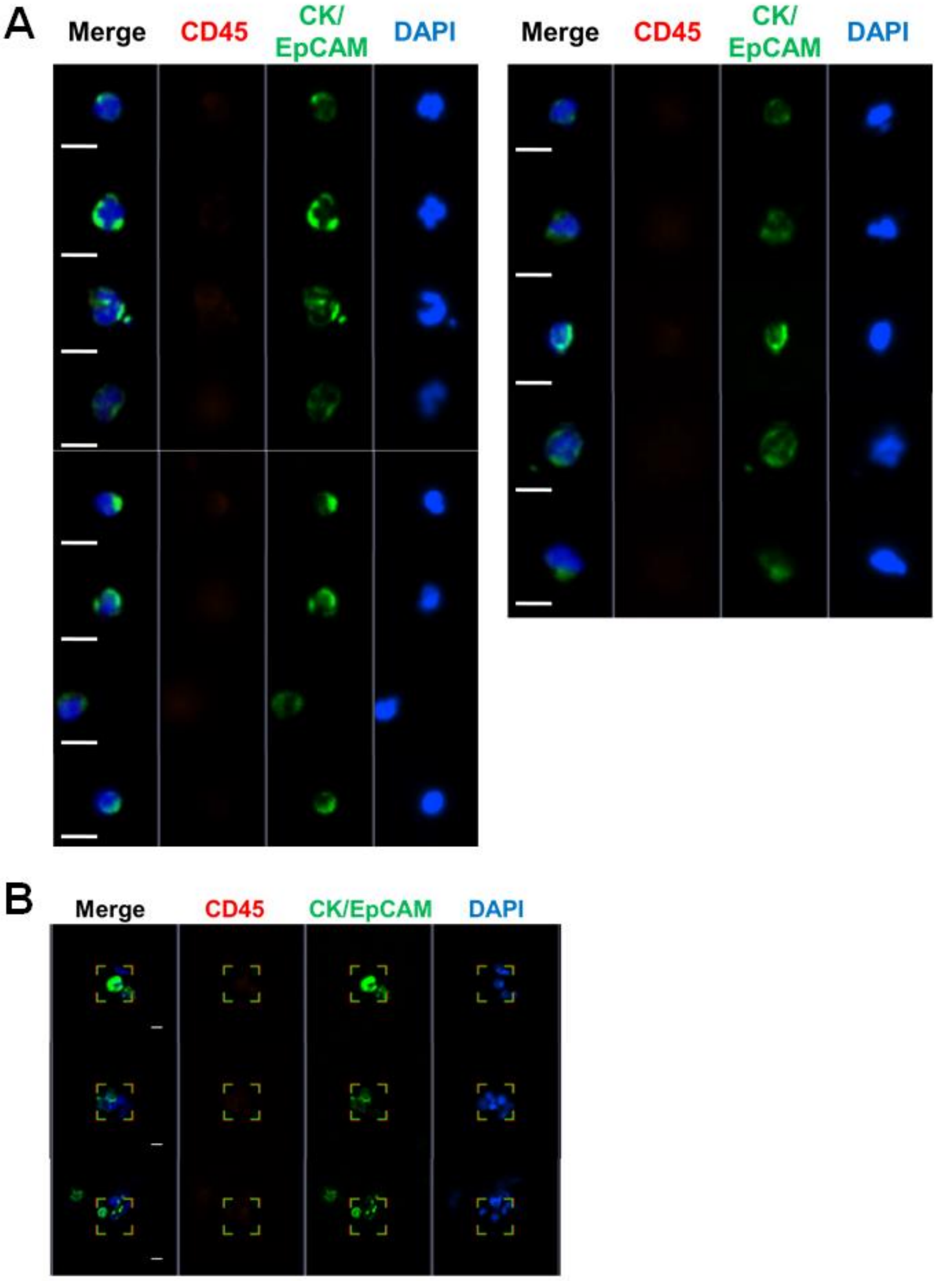

Figure S1. Fluorescence images of patient-derived CTCs. Representative examples of (A) patient-derived CTCs from two different NSCLC patients (Scale bar: 10 μm) and (B) CTC clusters from NSCLC patients (Scale bar: 8 μm). Merged images and three images from different fluorescent channels (DAPI, TRITC, and FITC) are shown. CTCs were defined as $DAPI^+/CD45^-/CK^+$ or $EpCAM^+$ cells.

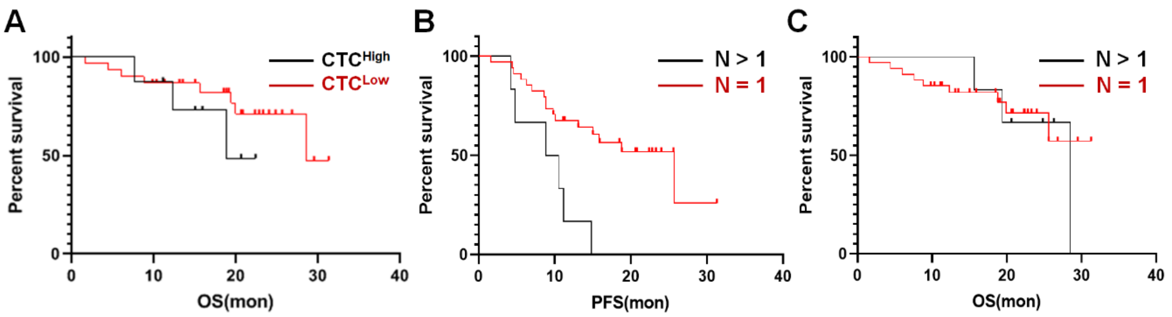

Figure S2. Correlation between CTC counts and treatment responses on CT scans. (A) Kaplan-Meier curves for OS between two patient groups according to the cutoff baseline CTC count (66 CTCs/7.5 mL). (CTC^{Low}: n = 30, median OS: 18.04; CTC^{High}: n = 8, median OS: 15.46, log-rank p = 0.3501; samples from two patients had a clogging issue.) **(B)** Six of 40 patients (15%) did not respond well to their first choice of therapy and had to change the treatment option more than once, resulting in a significantly poorer PFS than that of patients who responded well to the first-choice drug. Kaplan-Meier curves for PFS between two patient groups according to the number of treated drugs. (N = 1: n = 34, median PFS: 25.70, N > 1: n = 6, median PFS: 9.65, log-rank p = 0.0039). **(C)** Kaplan-Meier curves for OS between the two patient groups according to the number of treated drugs, demonstrating no significant difference. (N = 1: n = 34, median OS: undefined, N > 1: n = 6, median OS: 28.50, log-rank p = 0.7518).

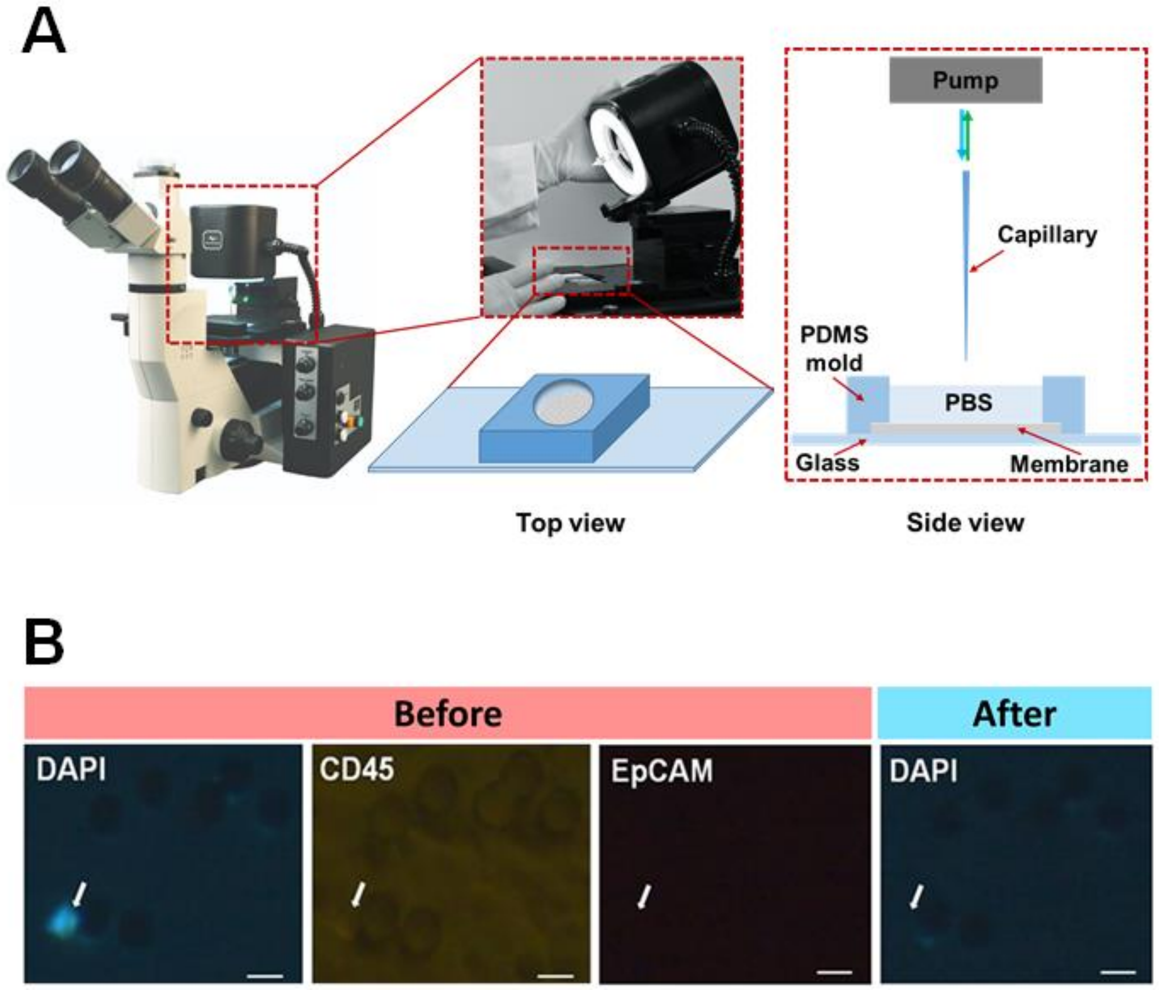

Figure S3. Procedures for single cell picking. (A) Schematic image of single cell picking. The membrane was separated from the disc and mounted onto the glass with the PDMS reservoir containing PBS. Using a capillary connected with a pump, a single cell was picked by the vacuum-assisted method. **(B)** Fluorescence images of a cell before and after picking from the membrane. DAPI+/CD45- stained cells are candidates for analysis. Image set of DAPI, CD45, and EpCAM before picking showing the target cells, and the other DAPI image shows the same spot after picking. (Scale bar: 10 μm).

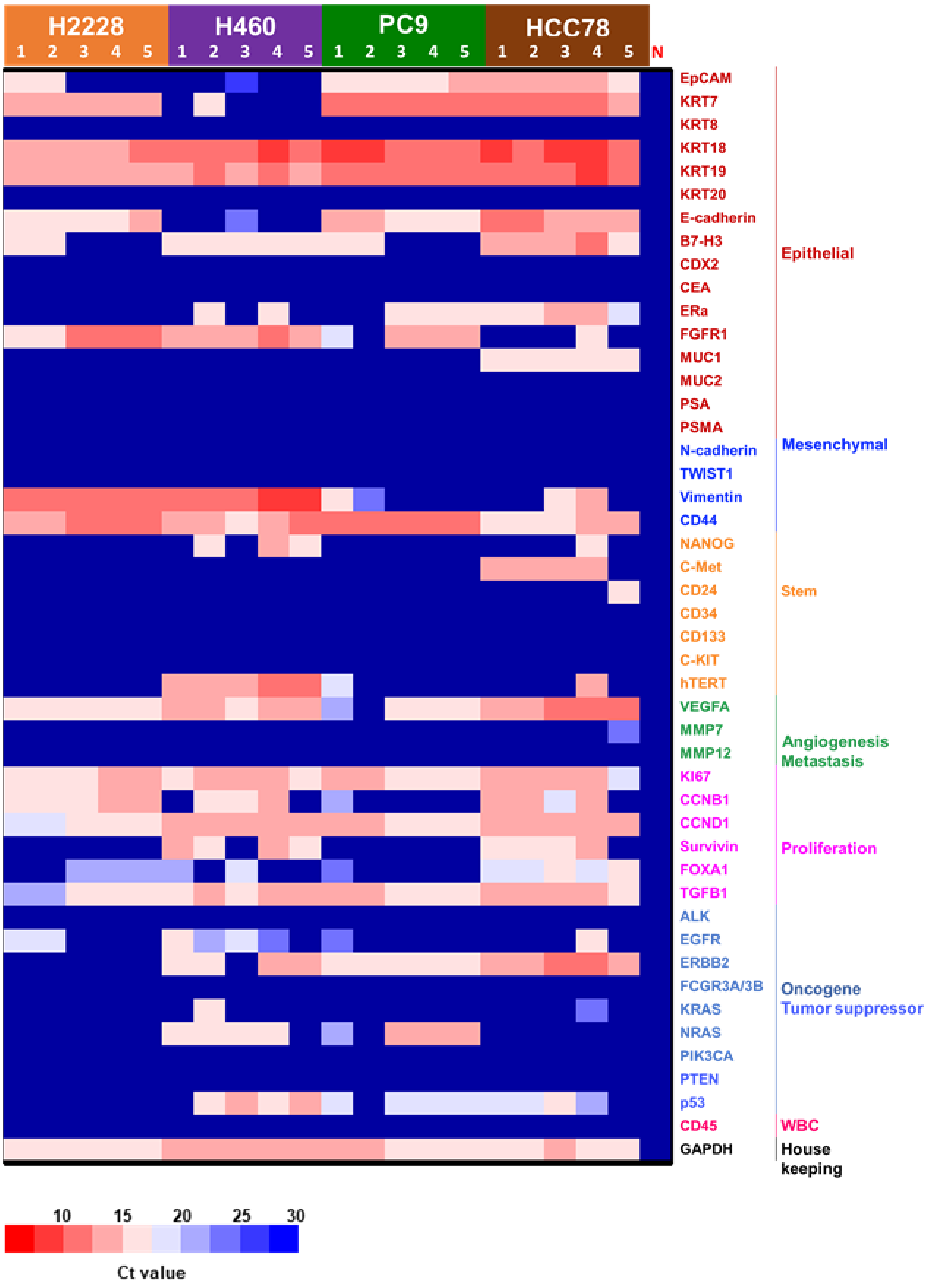

Figure S4. Single-cell real-time PCR using various lung cancer cell lines. Single-cell mRNA expression of four lung cancer cell lines, H2228, H460, PC9, and HCC78, determined using BiomarkHD qRT-PCR. After picking individual cells captured on the membrane by a cell-picker,

RNA was extracted and preamplified for single-cell mRNA expression analysis. Ct ≤ 30 with a Fluidigm real-time PCR analysis software quality threshold of 0.65 was considered for the analysis. Columns and rows show individual cells and the target genes assayed, respectively. N means no template control.

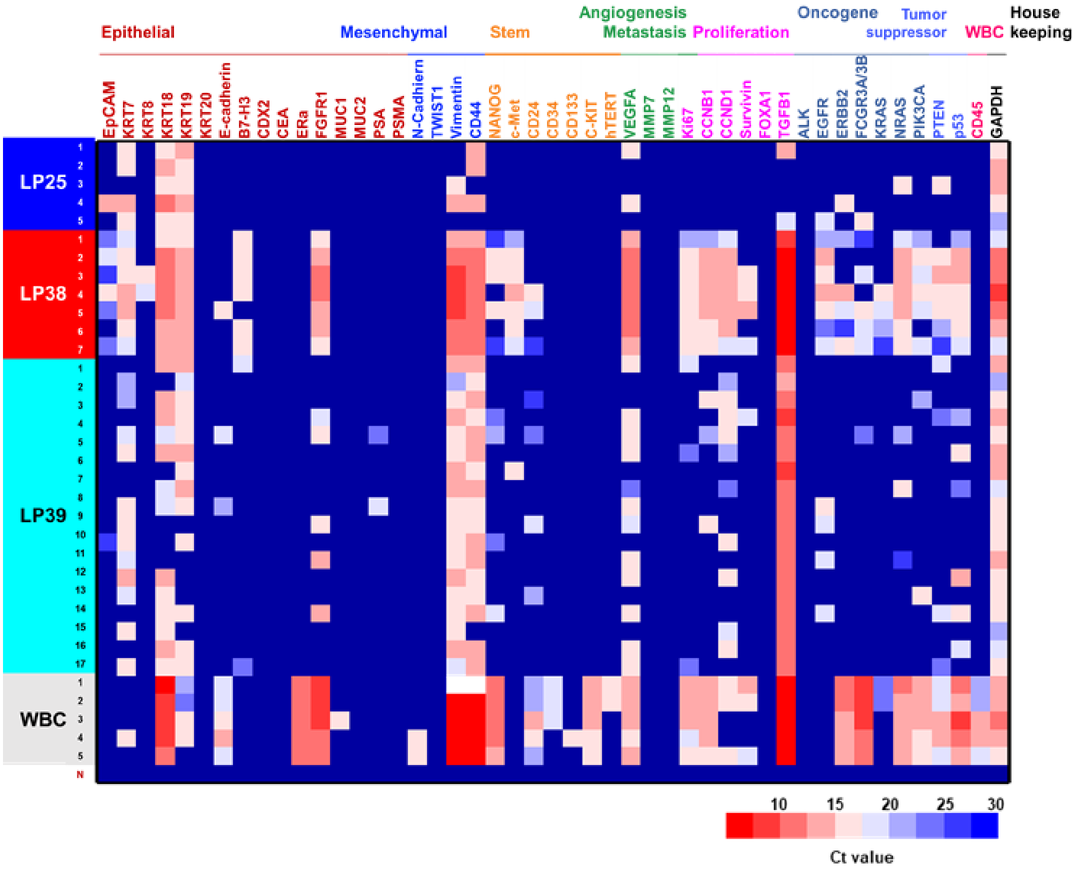

Figure S5. Single-cell real-time PCR using patient-driven CTCs from baseline samples and white blood cells. Single-cell mRNA expression of CTCs from three different patients at baseline (LP25, LP38, and LP39) determined using BiomarkHD qRT-PCR with five WBCs. Columns and rows show individual cells and the target genes assayed, respectively. N means no template control.

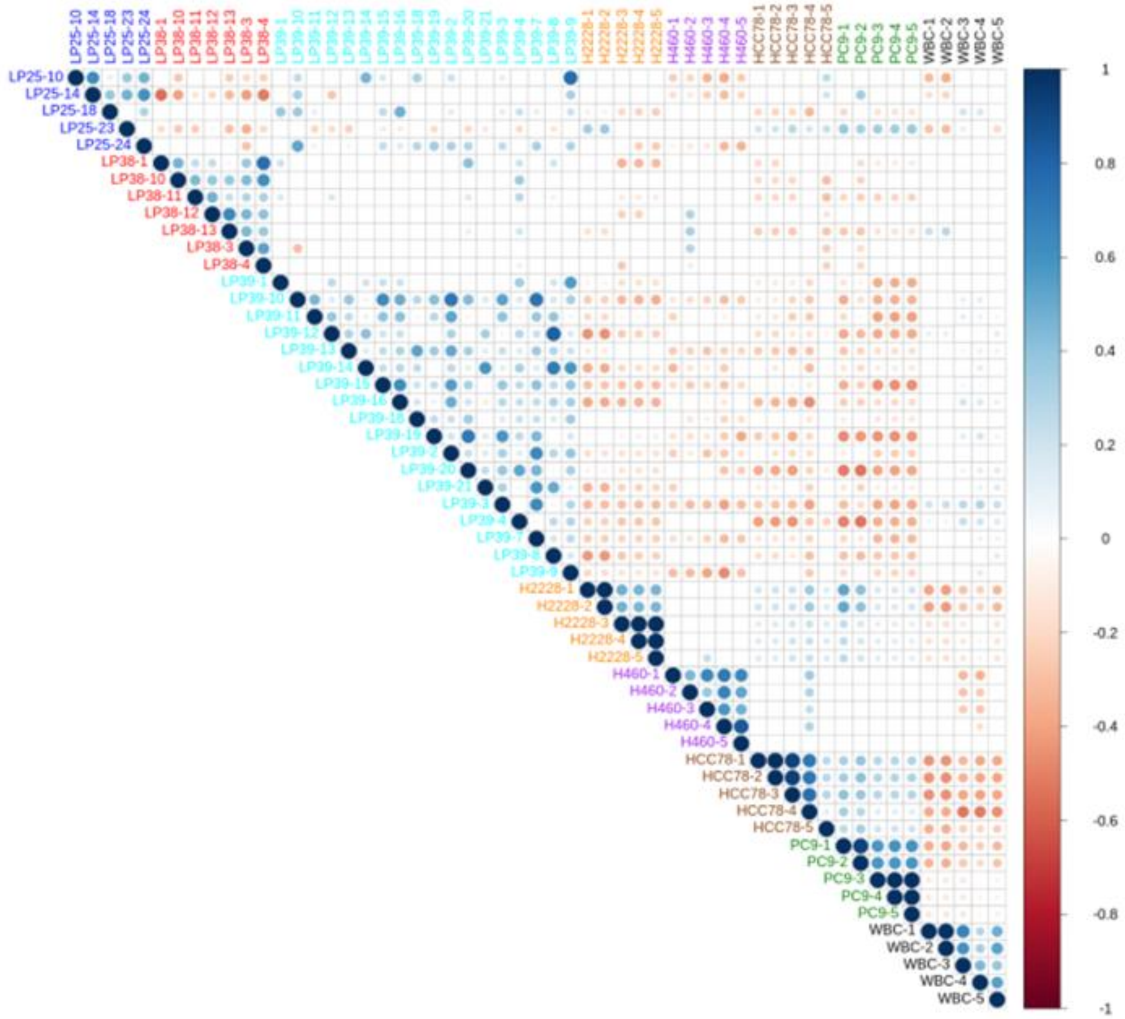

Figure S6. **Correlation matrix plots of correlations among 20 cells from four different cancer cell lines, five WBCs, and 30 patient-derived baseline CTCs isolated from three different patients.** Spearman correlation coefficients for sample characteristics. High and low similarity is indicated with blue and red color based on the scale bar, respectively. The circle size represents the magnitude of the correlation. P-values in this correlation analysis were derived using the *Cor.mtest* function in R. Bonferonni correction was applied to P-values to account for multiple testing in the rank correlation matrix.

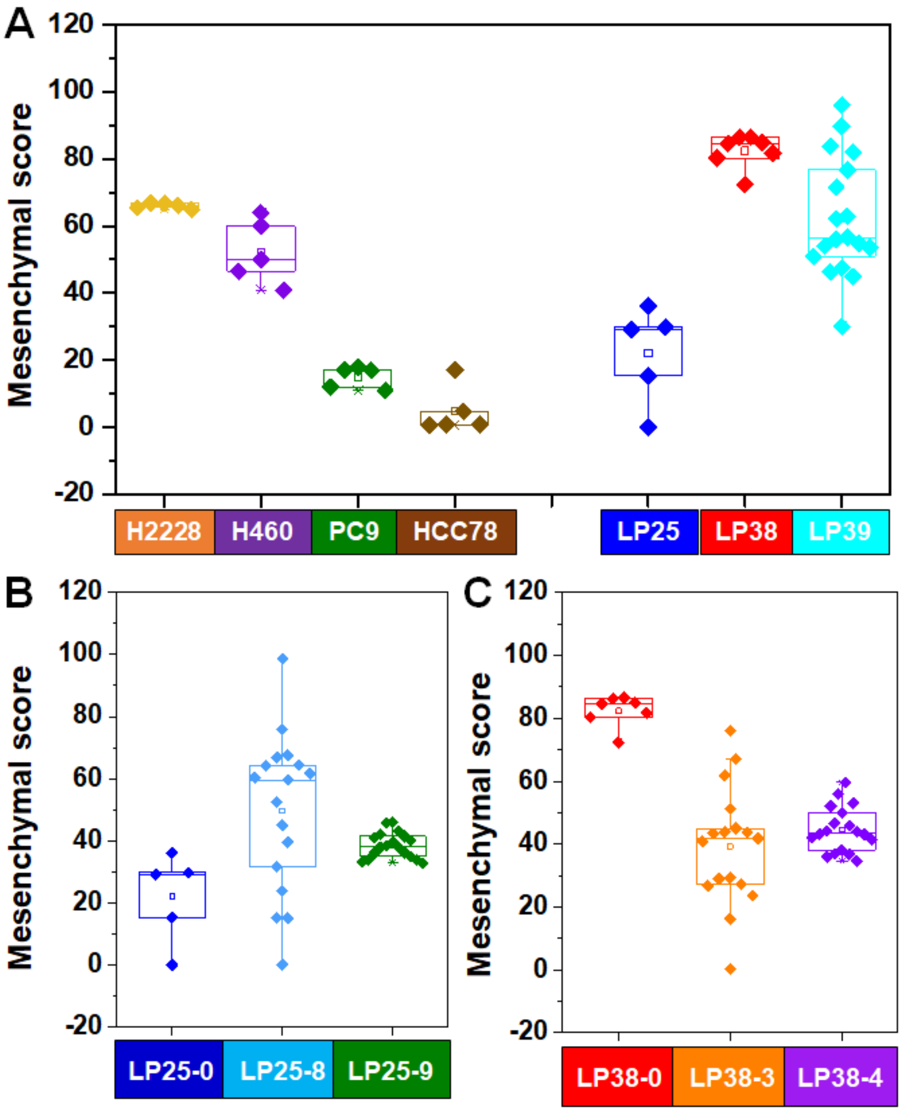

Figure S7. Summary of mesenchymal scores. (A) Distribution of mesenchymal scores from each sample according to the EMT characterization among four NSCLC cell lines and three NSCLC patients. Mesenchymal scores of single CTCs from patients **(B)** LP25 and **(C)** LP38 during TKI treatment.

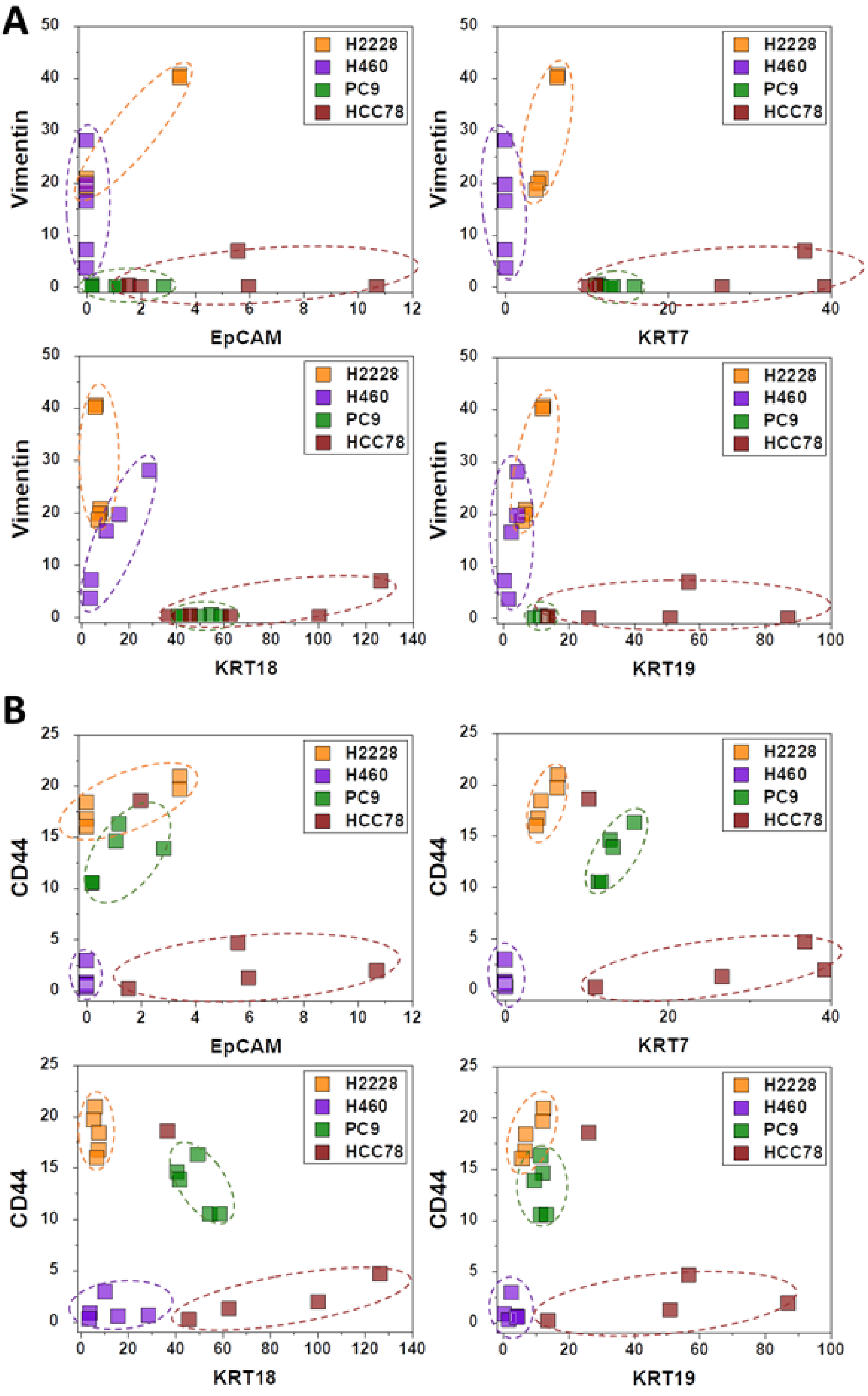

Figure S8. Correlation of marker expression among four NSCLC cell lines. (A) Vimentin expression of five cells from each cell line according to the expression of epithelial markers EpCAM, KRT7, 18, and 19, respectively. **(B)** CD44 expression of five cells from each cell line according to the expression of epithelial markers EpCAM, KRT7, 18, and 19, respectively.

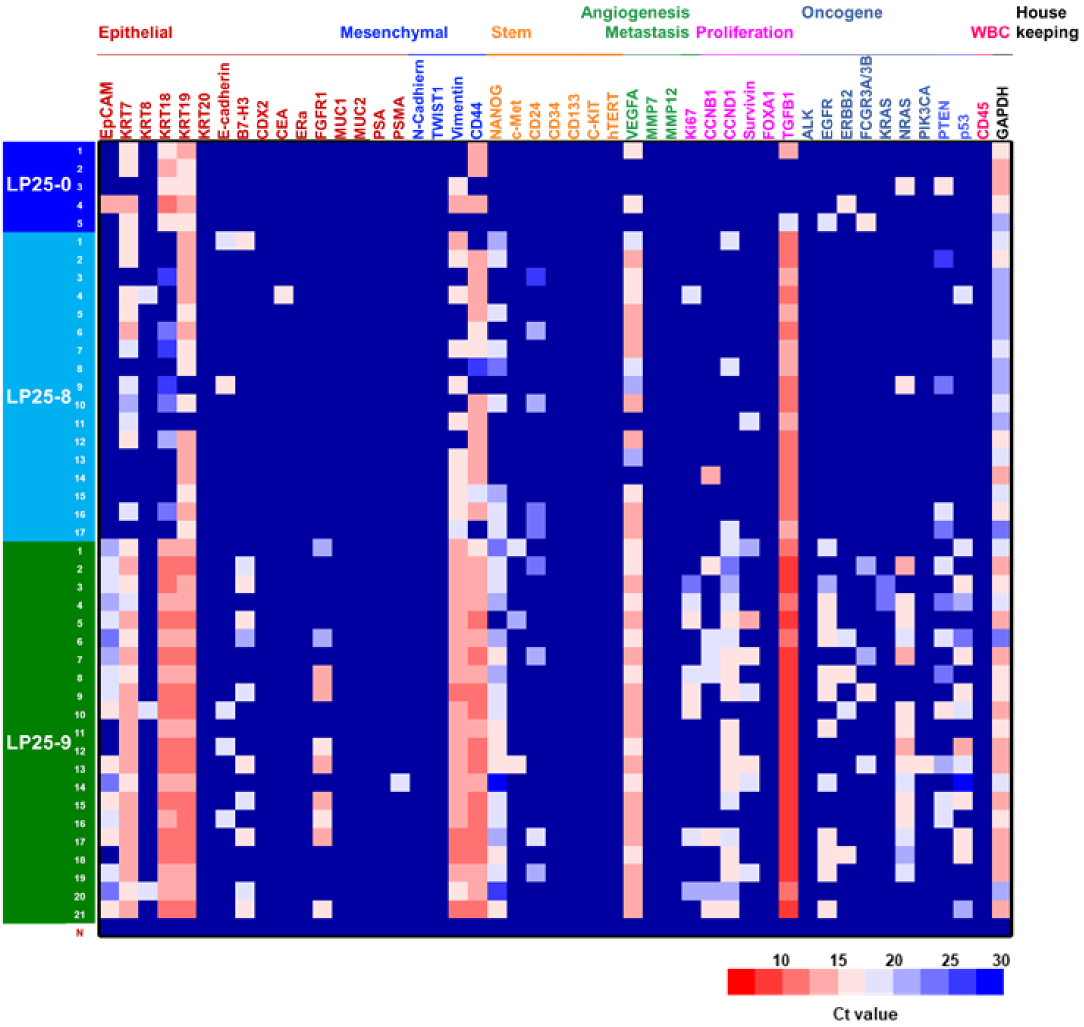

Figure S9. Single-cell real-time PCR using CTCs from patient LP25 at various time points. Single-cell mRNA expression was determined using BiomarkHD qRT-PCR. Columns and rows show individual cells and the target genes assayed, respectively. N means no template control.

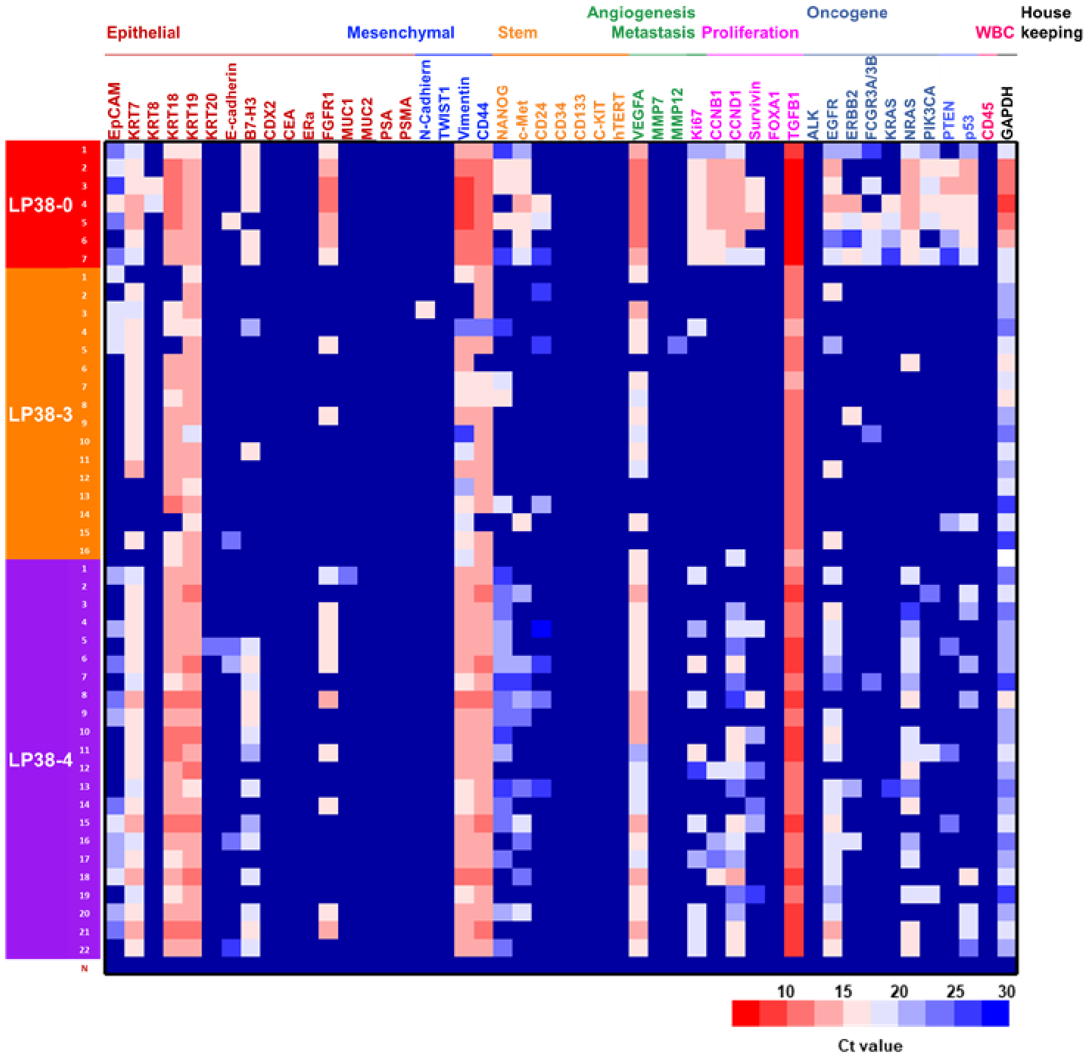

Figure S10. Single-cell real-time PCR using CTCs from patient LP38 at various time points. Single-cell mRNA expression was determined using BiomarkHD qRT-PCR. Columns and rows show individual cells and the target genes assayed, respectively. N means no template control.

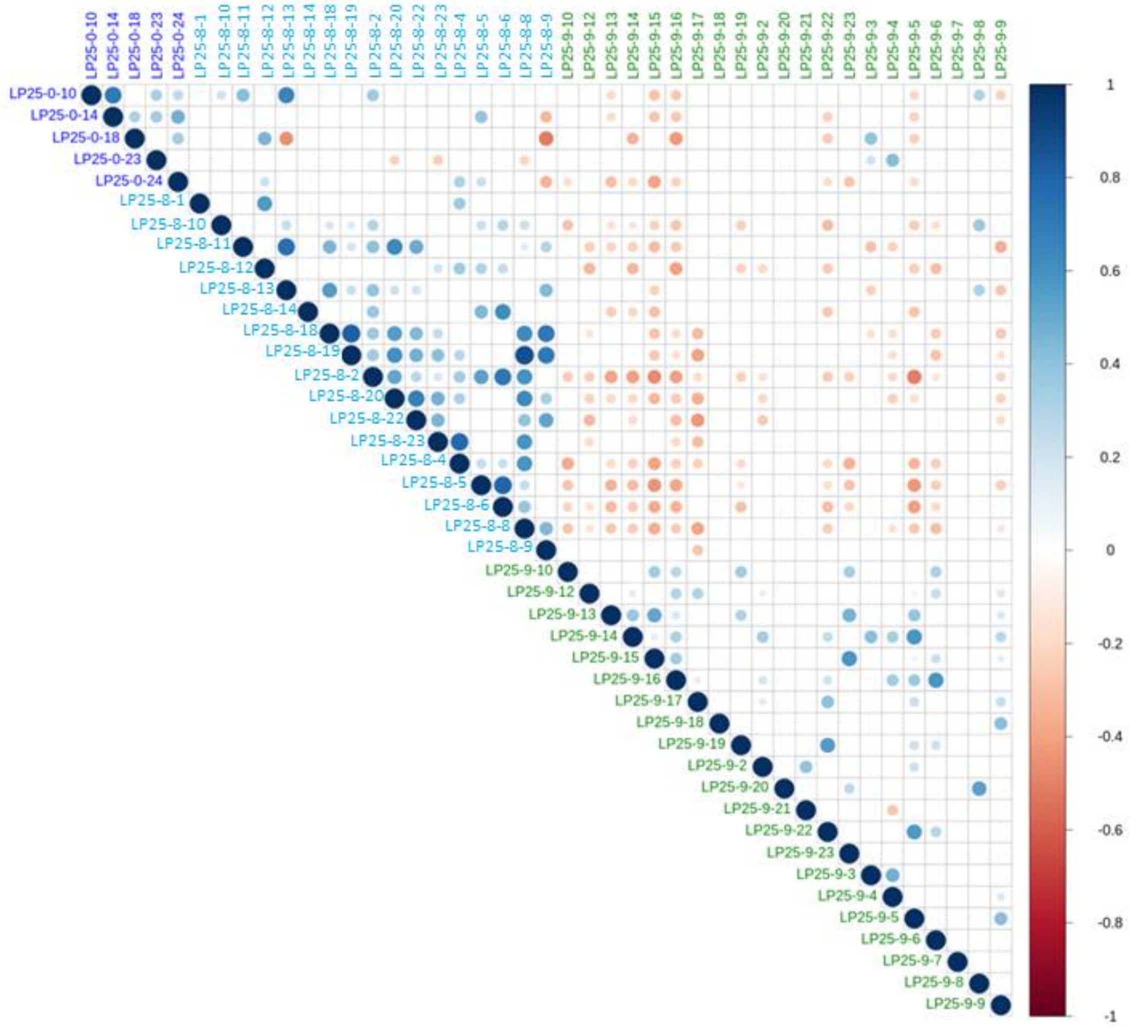

Figure S11. Correlation matrix plots of CTCs before and after treatment in patient LP25. Spearman correlation coefficients for sample characteristics. High and low similarity is indicated with blue and red color based on the scale bar, respectively. The circle size represents the magnitude of the correlation.

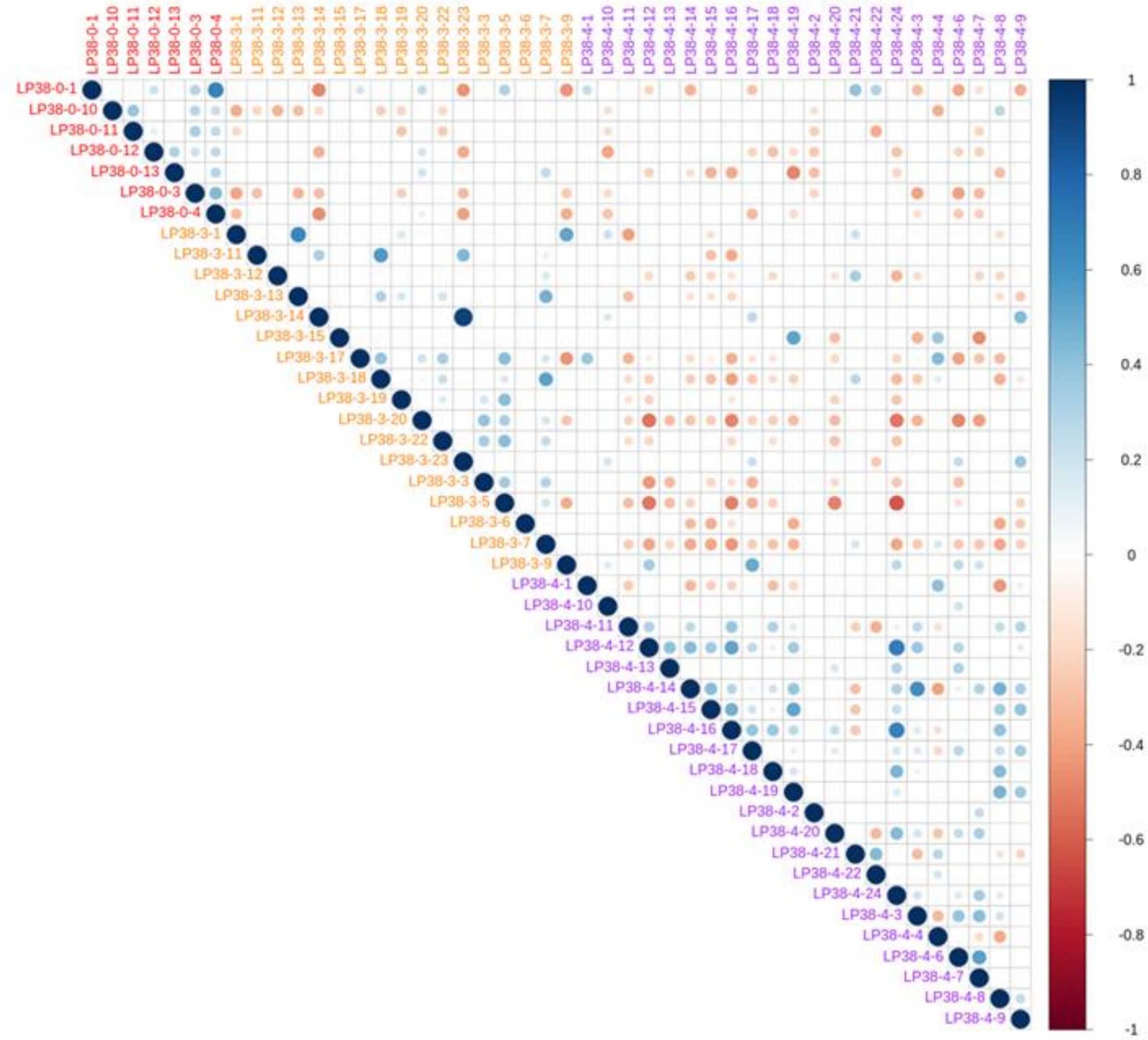

Figure S12. Correlation matrix plots of CTCs before and after treatment in patient LP38. Spearman correlation coefficients for sample characteristics. High and low similarity is indicated with blue and red color based on the scale bar, respectively. The circle size represents the magnitude of the correlation.

Table S1. Genes used to profile single CTCs. 48.48 dynamic array microfluidic qRT-PCR chips (Fluidigm) used to confirm the expression of genes in all captured single cells.

ALK	Anaplastic lymphoma kinase, CD246
B7-H3	CD276
CCNB1	Cyclin B1
CCND1	Cyclin D1
CD133	CD133 antigen, prominin-1
CD24	CD24 antigen
CD34	CD34 molecule
CD44	CD44 antigen
CD45	PTPRC; protein-tryosine phosphatase, receptor-type, C; leukocyte-common antigen
CDX2	Homeobox protein
CEACAM5	Carcinoembryonic antigen-related cell adhesion molecule 5; carcinoembryonic antigen (CEA)
c-MET	MET protooncogene; hepatocyte growth factor receptor (HGFR)
E-cadherin	CDH1; E-cadherin; liver cell adhesion molecule (LCAM)
EGFR	Epidermal growth factor receptor; HER1; ERBB1
EpCAM	Epithelial cellular adhesion molecule; tumor-associated calcium signal transducer (TACSTD1)
ERa	Estrogen receptor 1; estrogen receptor, alpha (ER-a), ESR1
ERBB2	HER2; V-ERB-B2 avian erythroblastic leukemia viral oncogene homolog 2; NEU
FCGR3A/B	Low affinity immunoglobulin gamma Fc region receptor III, CD16a/b
FGFR1	Fibroblast growth factor receptor 1
FOXA1	Forkhead box A1; hepatocyte nuclear factor 3-alpha (HNF3A)
GAPDH	Glyceraldehyde-3-phosphate dehydrogenase
hTERT	Telomerase reverse transcriptase
Ki67	MKI67, marker of proliferation Ki-67
KIT	Proto-oncogene c-Kit, CD117
KRAS	K-ras, KRAS proto-oncogne
KRT18	Kerain 18; cytokeratin 18
KRT19	Keratin 19
KRT20	Keratin 20
KRT7	Keratin 7
KRT8	Keratin 8; cytokeratin 8
MMP12	matrix metalloproteinase-12
MMP7	matrix metalloproteinase-7
MUC1	Mucin 1, transmembrane
MUC2	Mucin 2
NANOG	Nanog homeobox
N-cadherin	CDH2; neural cadherin (NCAD)
NRAS	Neuroblastoma RAS viral oncogene homolog
p53	Tumor protein p53, transformation-related protein 53(TRP53)
PIK3CA	phosphatidylinositol-4,5-bisphosphate 3-kinase, catalytic subunit alpha

PSA	Prostate-specific antigen
PSMA	prostate-specific membrane antigen, folate hydrolase 1(FOLH1)
PTEN	Phosphatase and tensin homolog
Survivin	Baculoviral IAP repeat-containing protein 5; apoptosis inhibitor 4 (API4); BIRC5
TGFB1	Transforming growth factor, beta-1
TWIST1	Twist, drosophila, homolog of 1
VEGFA	Vascular endothelial growth factor A
Vimentin	VIM
